# The thermal breadth of temperate and tropical freshwater insects supports the climate variability hypothesis

**DOI:** 10.1002/ece3.10937

**Published:** 2024-02-23

**Authors:** Beatrice S. Dewenter, Alisha A. Shah, Jane Hughes, N. LeRoy Poff, Ross Thompson, Ben J. Kefford

**Affiliations:** ^1^ Centre for Applied Water Science, Institute for Applied Ecology University of Canberra Canberra Australian Capital Territory Australia; ^2^ W.K. Kellogg Biological Station, Department of Integrative Biology Michigan State University East Lansing Michigan USA; ^3^ School of Environment and Science Griffith University Nathan Queensland Australia; ^4^ Department of Biology Colorado State University Fort Collins Colorado USA

**Keywords:** aquatic ectotherms, climate change, climate variability hypothesis, CT_max_, CT_min_, temperature variability, thermal breadth, thermal tolerance

## Abstract

Climate change involves increases in mean temperature and changes in temperature variability at multiple temporal scales but research rarely considers these temporal scales. The climate variability hypothesis (CVH) provides a conceptual framework for exploring the potential effects of annual scale thermal variability across climatic zones. The CVH predicts ectotherms in temperate regions tolerate a wider range of temperatures than those in tropical regions in response to greater annual variability in temperate regions. However, various other aspects of thermal regimes (e.g. diel variability), organisms' size and taxonomic identity are also hypothesised to influence thermal tolerance. Indeed, high temperatures in the tropics have been proposed as constraining organisms' ability to tolerate a wide range of temperatures, implying that high annual maximum temperatures would be associated with tolerating a narrow range of temperatures. We measured thermal regimes and critical thermal limits (CT_max_ and CT_min_) of freshwater insects in the orders Ephemeroptera (mayflies), Plecoptera (stoneflies) and Trichoptera (caddisflies) along elevation gradients in streams in temperate and tropical regions of eastern Australia and tested the CVH by determining which variables were most correlated with thermal breadth (*T*_br_ = CT_max_ − CT_min_). Consistent with the CVH, *T*_br_ tended to increase with increasing annual temperature range. *T*_br_ also increased with body size and *T*_br_ was generally wider in Plecoptera than in Ephemeroptera or Trichoptera. We also find some support for a related hypothesis, the climate extreme hypothesis (CEH), particularly for predicting upper thermal limits. We found no evidence that higher annual maximum temperature constrained individuals' abilities to tolerate a wide range of temperatures. The support for the CVH we document suggests that temperate organisms may be able to tolerate wider ranges of temperatures than tropical organisms. There is an urgent need to investigate other aspects of thermal regimes, such as diel temperature cycling and minimum temperature.

## Graphical abstract

We measured the critical minimum and maximum thermal tolerances of freshwater insects of the orders Ephemeroptera (mayflies), Plecoptera (stoneflies) and Trichoptera (caddisflies) along a temperate and a tropical elevational gradient in eastern Australia. Temperate species generally tolerated a wider range of water temperature than the tropical species, and thermal breadth was positively correlated with annual temperature range. Our findings support the climate variability hypothesis that long‐term exposure to high annual scale variability in temperature leads to the evolution of wide thermal tolerance.

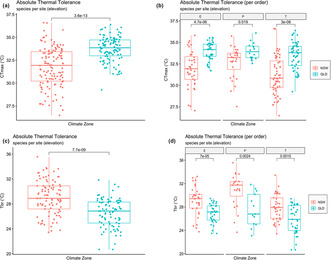

## 1 INTRODUCTION

Climate change is altering thermal regimes (means, variability and extremes) at multiple temporal scales across the planet (Coumou & Rahmstorf, 2012; Diffenbaugh et al., 2017; Duan et al., 2017; Geerts, 2003; Qian & Zhang, 2015; Thorne et al., 2016; Wallace & Osborn, 2002; Wang & Dillon, 2014). Alteration in thermal regimes affects organisms via multiple mechanisms, with important implications for vulnerability to climate change (see review by Kefford et al. (2022)). Most studies have focussed on species response to only one aspect of climate change, most frequently changes in mean temperature, in order to make predictions about vulnerability to warming (Bozinovic et al., 2011; Colinet et al., 2015; Deutsch et al., 2008; Dowd et al., 2015; Estay et al., 2014; Huey et al., 2012; Sinclair et al., 2016; Sunday et al., 2014; Thompson et al., 2013; Vasseur et al., 2014). Few, however, have attempted to link physiological traits to thermal variability (Morash et al., 2018; but see Polato et al., 2018; Shah, Funk, & Ghalambor, 2017; Shah, Gill, et al., 2017), which can have a much stronger effect on organismal response than mean temperature change (Bernhardt et al., 2018). Thus, there is a pressing need to understand how organisms respond to a range of components of thermal regimes in order to develop a more complete understanding of the likely impacts of changing climate (Kefford et al., 2022; Sheldon et al., 2018). For example, high intra‐specific variation in thermal tolerance would tend to protect species' populations from warming, whereas high inter‐specific variation in thermal tolerance may tend to protect community functions, assuming redundancy between species, from warming, as some species would have greater thermal tolerance change (Bolnick et al., 2011; Moran et al., 2016; Pacifici et al., 2015), although phenotypic plasticity or acclimation may also be important.

The observation of less annual scale variability in temperature in the tropics relative to temperate regions and the effect of changes in this variability across latitudes on biota can be traced back to Alexander von Humboldt in the 1800s and were later further developed (e.g. Dobzhansky, 1950). Subsequently, Janzen (1967) applied some of these ideas to elevation gradients and developed what we are calling the ‘climate variability hypothesis’ (CVH), which generates a clear set of testable predictions about how thermal tolerance will change across elevational gradients in temperate versus tropical mountains. Specifically, the hypothesis predicts that the range of temperatures that organisms can tolerate (often measured as thermal breadth *T*_br_) is positively correlated with the annual temperature range an organism has experienced over evolutionary time. Thus, organisms that have evolved in environments with relatively narrow annual temperature ranges, such as the tropics, should in general have narrower thermal breadths than organisms that have evolved in high‐latitude areas with wide annual temperature ranges (Ghalambor et al., 2006; Janzen, 1967; Polato et al., 2018; Sheldon et al., 2018; Stevens, 1989). Although there is empirical support for the CVH (e.g. see reviews by Ghalambor et al., 2006; Sunday et al., 2011), including with freshwater insects (Polato et al., 2018; Shah, Funk, et al., 2017; Shah, Gill, et al., 2017) the subject of this paper, patterns are not universal. For example, there is a steeper relationship between *T*_br_ and latitude in the Northern than in the Southern Hemisphere (Sunday et al., 2011). General hypotheses, like the CVH, are predicted to apply across all continents and in both hemispheres and it is important that their generality is tested.

In addition to the annual temperature range, there are other aspects of thermal regimes that may be important in shaping the physiological tolerances of species. For instance, Payne and Smith (2017) proposed that the thermodynamics of high temperatures in the topics constrain an organism's abilities to tolerate a wide range of temperatures (see also Dillon et al., 2010; Gillooly et al., 2006) as a contrasting mechanism to the CVH (Janzen, 1967). Payne and Smith's (2017) hypothesis predicts that tropical organisms living in cool conditions at relatively high elevations would tend to have wider *T*_br_ than tropical organisms living in warm conditions at relatively low elevations. Although proposed as an alternative, Payne and Smith's hypothesis is not necessarily mutually exclusive of Janzen's CVH and *T*_br_ could plausibly be influenced both by evolution and thermodynamic constraints (Kefford et al., 2022). Thermal tolerances and thus *T*_br_ may also be shaped (partly) by annual minimum temperature (Marshall & Sinclair, 2012), acute temperature change (Colinet et al., 2015; Dowd et al., 2015; Vázquez et al., 2017) and diel temperature variability (Chan et al., 2016; Gilchrist, 1995; Ørsted et al., 2022). It is still unclear which of these aspects of thermal regimes shape thermal breadth (Kefford et al., 2022).

However, it is clear that body size and taxonomic associations of ectotherms are important covariates for temperature tolerance and thus thermal breadth (Leiva et al., 2019; Peralta‐Maraver & Rezende, 2021). Body size at maturity for ectotherms has frequently been shown to decrease with increasing temperature (Atkinson, 1994; Kingsolver & Huey, 2008; Sweeney et al., 2018; Sweeney & Vannote, 1978). Temperature appears to affect body size via two mechanisms (Kingsolver & Huey, 2008). Firstly, phenotypic plasticity tends to result in individuals reared at cool temperatures being larger than individuals reared at warmer temperatures. Secondly, adaptive evolution can result in populations and species in cool environments tending to be larger than those in warm environments (Bergmann's rule). In both mechanisms, examples of opposite relationships also occur (Kingsolver & Huey, 2008). While hypotheses have been proposed to explain why organisms would tend to be smaller in cooler than warmer environments, no hypothesis is well supported, see Kingsolver and Huey (2008) for details. A key issue is that body size is associated with changes in tolerance to both cold and warm temperatures, complicating the relationship between body size and thermal breadth (Leiva et al., 2019). Thermal tolerance has been shown to vary substantively among taxa with different evolutionary histories (Bennett et al., 2021).

A related hypothesis to the CVH is the climate extreme hypothesis (CEH) (Pither, 2003; Sunday et al., 2019) which proposes that relatively rare extreme climatic events determine an organism's tolerance to both cold and warm temperatures. The CEH predicts a positive relationship between an organism's cold tolerance and minimum temperatures experienced, and between organism's heat tolerance and maximum temperatures experienced. The CVH focusses on temperature variability over the annual cycle and not minimum or maximum extremes per se. For example, the CVH predicts that organisms experiencing minimum and maximum temperatures of 0°C and 20°C, respectively, would have the same *T*_br_ as those experiencing minimum and maximum temperatures of 10°C and 30°C, respectively. The CEH predicts in these two scenarios organisms would have different tolerances to cool and warm temperatures.

Shah, Gill, et al. (2017) compared the thermal tolerances of freshwater insects in temperate North and Equatorial America, finding support for the CVH (although they did not consider the CEH). However, the global and taxonomic generality of their findings has not been determined. In addition, the extent to which aspects of thermal regimes other than annual thermal range contribute to thermal tolerances has not been investigated in any study. Here, we experimentally determined thermal tolerance and calculate *T*_br_ of freshwater insects collected from temperate (36° S) and tropical (17° S) streams with markedly different temperature regimes in eastern Australia. We had three specific aims:

1. To test the prediction of the CVH in the Southern Hemisphere that a larger annual temperature range would be positively correlated with thermal breadth (*T*_br_).
2. To test the extent to which biological variables (taxonomic order and body size) and an expanded number of environmental temperature variables (annual minimum, maximum, mean temperature, diel temperature range) are correlated with *T*_br_.
3. To compare intra‐ and inter‐specific variation in *T*_br_ to assess how biota from temperate and tropical regions may vary in their sensitivity to warming.
4. To test the predictions of the CEH.

## 2 MATERIALS AND METHODS

**FIGURE 1:**
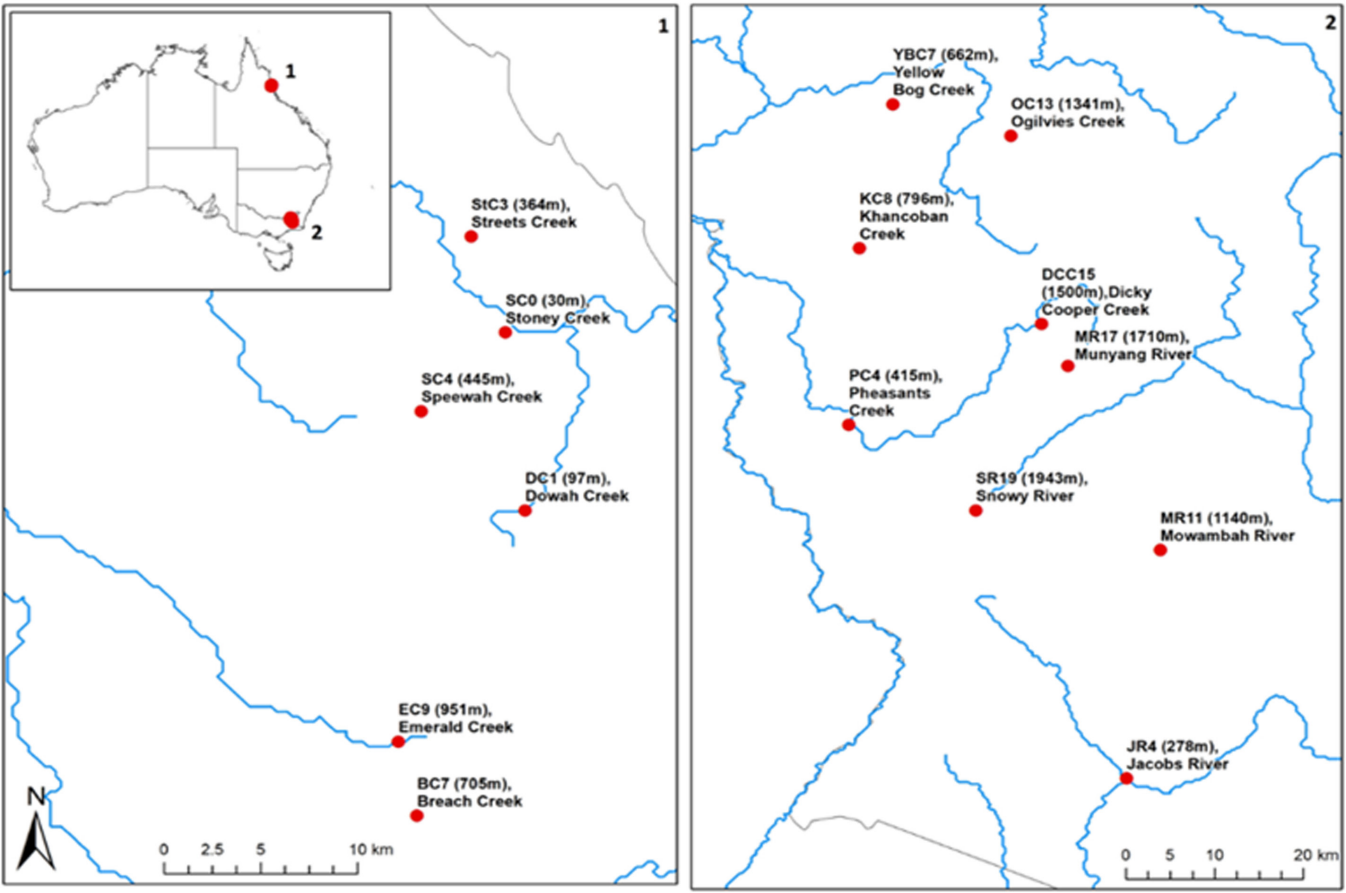
Map of locations of collection sites in (1) tropical (northern Queensland, QLD) [left panel] and (2) temperate (southern New South Wales, NSW) [right panel].

We experimentally determined CT_min_ and CT_max_ for multiple populations of stream insects collected along elevation gradients in tropical northern Queensland (QLD) and temperate southern New South Wales (NSW), Australia (Figure 1a). We measured CT_min_ and CT_max_ in multiple morphospecies from the insect orders Ephemeroptera (E, mayflies), Plecoptera (P, stoneflies) and Trichoptera (T, caddisflies), as these orders are important components of aquatic biodiversity in montane regions (Jacobsen et al., 2012).

### 2.1 Site information

Nine temperate sites were selected in NSW, in the Snowy Mountain Bioregion (36° S, 148° E) between 278 and 1943 m above sea level (asl) (Figure 1; see Table S1 for site locations). Six tropical study streams were selected in the Wet Tropics Bioregion in QLD (17° S, 145° E), between 30 m and 952 m asl. All sites were in or adjacent to National Parks with minimal upstream anthropogenic disturbance, including from livestock and feral grazers. To control for confounding effects of habitat and stream size, sites were within riffles in small tributaries (1st–3rd order) that drained to a larger, mainstem stream. To ensure that streams were comparable except for their thermal regimes, a range of potential confounding variables were measured including water depth, stream flow rate (FH950 Handheld Flow Meter; HACH, USA), water pH, dissolved oxygen, electrical conductivity and turbidity (U‐52 MultiParameter Water Quality Meter; HORIBA), alkalinity (field titration), total nitrogen, ammonia, nitrite, nitrate (USEPA Nessler, USEPA Diazotization and the Cadmium Reduction Method, TPS 90 FL Field Lab Analyser; HACH, USA) and total phosphorus (Hach Orthophosphate Test Kit; PO‐14; HACH, USA).

### 2.2 Quantification of stream temperature regimes

We used 12 months of data (April 2019 to April 2020 in NSW and July 2019 to July 2020 in QLD) from submerged temperature loggers (TidbiT MX Temp 400; HOBO, USA) to record water temperature (°C) at 15‐min intervals at all sites. At the 30 and 450 m sites in QLD the data loggers failed. We consequently replaced the temperature data of the 445 m site with the temperature data of a nearby site at 482 m asl (Table S1).

To characterise thermal regimes at each site we calculated, the annual absolute minimum and maximum temperatures, that is, the lowest/highest temperature observed over the 365 days period (hereafter annual minimum and maximum temperatures), mean temperatures, diel temperature variation, seasonal temperature range (as the maximum temperature during the season we were measuring thermal tolerances (temperate = autumn; tropical = dry season) minus the minimum temperature during this season) and annual temperature range (as the absolute hottest temperature minus the absolute coldest measured temperature over the 365 days). To calculate the diel temperature variation, we first calculated the diel temperature range (daily maximum minus daily minimum) for every 365 days, then calculated the mean of these ranges and used this to represent diel temperature variation.

### 2.3 Aquatic macroinvertebrate collection, transport and acclimation

Aquatic macroinvertebrates were collected between March and May (autumn) 2019 in temperate NSW, and between July and August (dry season) 2019 in tropical QLD, using a D‐frame kick net (mesh size 500 μm). Individuals used for trials were live‐picked in the field and provisionally identified based on shared morphological features. In some cases, morphospecies were defined based on apparent morphological differences not strictly following the identification resources (see Tables S3 and S4, for a list of morphospecies). Taxa used in the trials were from four (NSW) and three (QLD) Ephemeroptera families, eight (NSW) and 10 (QLD) Trichoptera families and four (NSW) and two (QLD) Plecoptera families. However, 63% of EPT families occurred in both NSW and QLD at multiple elevations, thereby allowing for comparisons between regions largely unconfounded by phylogeny. Nevertheless, the mixed‐effect modelling we conducted (see below) accounted for the potential phylogenetic difference between the temperature and tropical locations. We separated the different taxa and transported them (between 1 and 3 h) to a field laboratory in 2.3 L plastic containers (food safe; BPA free) containing oxygenated and cooled stream water, and a fixed piece of 500 μm mesh for the organisms to cling to. In the laboratory, we transferred each taxon into clean containers, each holding two disinfected stones as hiding spaces to minimise stress, 1 L filtered stream water with high oxygen levels and moderate current generated by submerged air pumps (AP180; AQUA Syncro, China).

To standardise testing conditions across individuals and taxa, we used a standard acclimation period (48 h at 17°C, 12:12 d/n) (García‐Robledo et al., 2016) (Allen et al., 2016). The 17°C temperature was chosen as this was the expected mean temperature of a mid‐elevation stream in tropical QLD during our testing period. To ensure all animals were tested in a post‐absorptive state, we did not feed organisms during acclimation (Rezende et al., 2011; Shah, Gill, et al., 2017).

### 2.4 Determination of critical thermal limits

We followed Shah, Gill, et al. (2017) dynamic method to measure CT_min_ and CT_max_. Briefly, after acclimation, insects were randomly assigned to testing for either CT_min_ or CT_max_. We tested CT_min/max_ by placing one individual into a mesh beaker inside a water bath, with water pumped to create a flow and aerated for oxygenation. We ramped the water temperature at approximately +0.25°C/min (for CT_max_) or −0.25°C/min (for CT_min_) using a calibrated temperature controller (Unisat Heater Circulator – 2000 W, Thermoline Scientific, Australia) and a cooling coil (TIC‐400 Immersion Cooler, Thermoline Scientific, Australia; supported by frozen river water below 5°C). This ramping rate is considered too slow for thermal shock and too fast for acclimation to occur (Shah, Gill, et al., 2017) and allowed us to determine differences in CT_max_ between tropical and temperate ectotherms (Allen et al., 2016). We recorded the water temperature (°C) as the CT_min_ and CT_max_ value when individuals showed impaired locomotor performance, that is, loss of righting response, and loss of the ability to cling to the mesh beaker, often preceded by body spasms. A few cold‐tolerant individuals showed no such changes at slightly below 0°C at which time the experiment was terminated due to ice forming. In these cases, CT_min_ was recorded as the temperature at which the experiment was terminated, despite the possibility that individuals may survive freezing. Immediately following trials, insects were placed back at their acclimation temperature (17°C) to recover for approximately 45 min. Most (93.7%) individuals made a full recovery, that is, returned to normal movement and swimming activity. Data from these individuals were used in our analyses. Individuals who died, moulted or emerged during recovery were excluded from the data set, as were individuals who showed visible parasite infestation by dipterans (*Symbiocladius* pupa (Chrionomidae)). All test individuals were preserved and subsequently identified to the lowest taxonomic level possible (morphospecies, otherwise genus) (see Text S1 for a list of identification resources used, see Tables S3 and S4, for a list of morphospecies, their family and order (E, P or T) and the numbers of individuals tested for CT_min_ and CT_max_). Genetic data indicate that there are undescribed and crypto species present (Jollene K.A. Fraser, University of Canberra, unpublished data). We measured the head width of individuals to the nearest 0.001 mm with a Leica Application Suite v.3.8.0 (Leica Microsystem CMS GmbH, Switzerland) as a correlate of total body size (Benke et al., 1999). The size of individuals when CT_max_ or CT_min_ was measured may be somewhat smaller than their size at maturity.

### 2.5 Calculation of thermal breadth

For each site where we had measurements of both CT_max_ and CT_min_ from the same morphospecies, we calculated thermal breadth (*T*_br_ = CT_max_ − CT_min_). For each morphospecies that were found at ≥2 sites, we calculated the grand mean and log‐transformed standard deviation of *T*_br_ over all elevations, separately for NSW and QLD. Following Nati et al. (2021), we used this log‐transformed standard deviation as an index of intra‐specific variability in *T*_br_. We then calculated the inter‐specific variability as the mean differences between these grand mean *T*_br_ between each combination of morphospecies within a climate zone.

### 2.6 Data analysis

Statistical analyses were conducted in R version 4.0.2 (R Core Team, Vienna). To test for an elevation temperature gradient, we performed an ANOVA for each latitude with site as a categorical variable to check if stream water temperature varied between sites at different elevations. To test the CEH (Pither, 2003; Sunday et al., 2019) ordinary least squares regression models were used to explain CT_min_ and CT_max_ based on the absolute minimum annual and absolute maximum annual, respectively, water temperatures. These models were fitted both for CT_min/max_ values from both climate regions and for each region separately.

We used linear mixed‐effect models (R package *lme4*; Bates et al., 2009) to investigate whether *T*_br_ changed with the annual temperature range as predicted by the CVH. Shah, Gill, et al. (2017) in their analysis used phylogenetic generalised least squares regression (PGLS). To test whether *T*_br_ varied in response to factors other than annual temperature range, we initially chose six thermal variables: annual temperature range, temperature range during sampling season, diel temperature variability and annual minimum, maximum and mean temperature. We used untransformed temperature data to retain the relationship between temperature regimes. We also included two biological variables (taxonomic order [Ephemeroptera, Plecoptera or Trichoptera] and mean body size [average head width in mm] per morphospecies) as predictors of *T*_br_ for each region. We tested body size for normal distribution with a Shapiro test and log‐transformed this variable. Strong covariation among predictor variables results in increasing variances around parameter estimates, which can lead to inaccurate interpretation of parameter estimate importance in multivariate linear regression (Dormann et al., 2013). We addressed this by performing a PCA with the temperature variables, which creates a correlation matrix using Pearson's *r*, to identify highly correlated variables, that is, with *r* ≥ .7 (Dormann et al., 2013) and eliminated redundant variables (see below).

The PCA determined that temperature variability at the diel scale, temperature range during our sampling season, as well as the annual minimum and mean temperature, were highly correlated with the annual temperature range (Table S2 and Figure S1). We, therefore used five fixed effects: annual temperature range because of its importance in the CVH (Janzen, 1967), annual maximum temperature because it was not strongly confounded with annual temperature range and because high temperatures have been hypothesised as an alternative mechanism to the CVH (Payne & Smith, 2017), the interaction between those two temperature variables, taxonomic order and the log‐transformed mean of body size for each morphospecies at each site. The interaction was included to test for a logical possible relationship which could have important effects, but for which we had no formal hypothesis. As they were not relevant to our main question, we included sampling site elevation (m asl) and nested taxonomic hierarchy (family, genera and species) as random effects in the model, to account for any local or elevation effects as well as phylogenetic relationships. The best‐fit linear mixed‐effect model was selected using the Akaike information criterion (AIC) using the *model.sel* function in the package *MuMIn* (version 1.43.17; Barton, 2009). The hierarchy of each predictor was given by the model summary and was plotted based on the estimates and standard errors. *R* ^2^ values for the two best‐fit models were generated with the *r2_nakagawa* function in the package *performance* (Lüdecke et al., 2021). A pairwise comparison between taxonomic orders was performed using the *emmeans* function in the package *emmeans* (version 1.7.3; Lenth et al., 2022).

We compared *T*_br_, of the tropical and temperate sites with the predictions based on the CVH. We used linear regression to test if *T*_br_ increased with increasing annual temperature range. We also tested if taxa from temperate NSW had a lower CT_max_ and lower CT_min_, as well as wider *T*_br_, than those from tropical QLD.

Additionally, although not connected with predictions of the CVH, we tested for higher intra‐ and inter‐specific variation in temperate than tropical regions in *T*_br_. High intra‐specific variation in *T*_br_ would potentially allow individuals of a species to persist following a change in climate (Franken et al., 2018; Herrando‐Pérez et al., 2019; Nati et al., 2021). High inter‐specific variation in *T*_br_ would suggest that although a change in climate might result in the loss of some species, other species would persist.

We conducted two‐way ANOVAs with fixed factors using latitude (temperate or tropical) and order (i.e. Ephemeroptera, Plecoptera or Trichoptera) and the interaction between these variables to test for differences in CT_min_ and CT_max_ between these categories to explain (any) differences in *T*_br_.

Boxplots were used to display relationships between the different indices per climate zone.

## 3 RESULTS

A total of 1476 (CT_min_) and 1683 (CT_max_) individual stream insects were assessed. These individuals came from 121 morphospecies, of which 40 were from the order Ephemeroptera, 22 were Plecoptera and 59 were Trichoptera (see Tables S3 and S4 for lists of morphospecies). Individual head widths ranged from 0.29 to 9.44 mm (mean = 1.36 mm, SD = ±0.77, *n* = 3159). The lowest CT_min_ observed (per site per morphospecies) was −0.1°C, and the highest CT_min_ was 10.3°C, with about 8% of CT_min_ values being <1°C. CT_max_ ranged from 26.5°C to 38.3°C.

Several environmental temperature parameters were highly correlated with each other. These included annual minimum, maximum and mean, diel temperature variability, seasonal temperature range (dry season in QLD, autumn in NSW) and the annual temperature range (*r* ≥ .7, Table S2 and Figure S1). Because predictions derived from the CVH primarily focus on the effect of annual temperature range on thermal physiology, we used this variable for our analyses. In addition, the annual maximum temperature was not strongly correlated with the annual temperature range (*r* = .33). Because high maximum temperatures are predicted to limit *T*_br_ (Payne & Smith, 2017), we retained this variable in the analysis.

### 3.1 Testing the climate variability hypothesis

**FIGURE 2:**
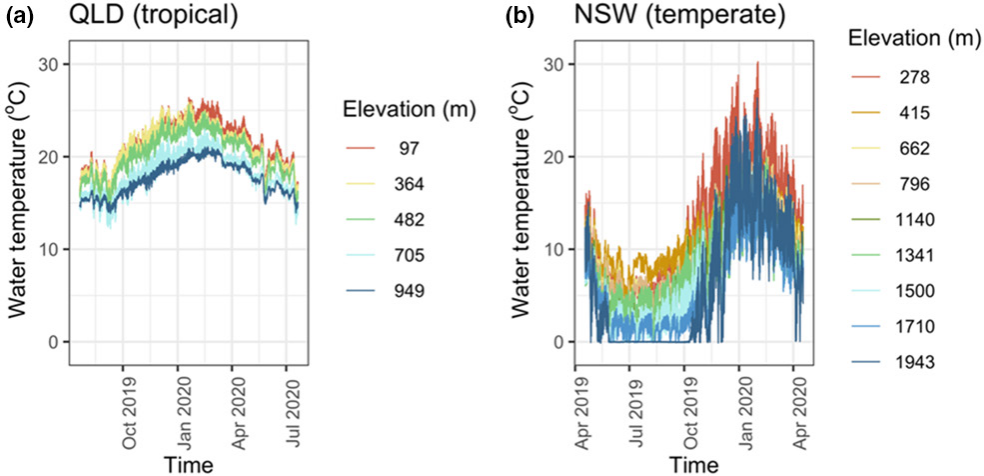
Water temperatures were recorded every 15 min per site over 365 days (a) in tropical (QLD) and (b) temperate (NSW) [right panel]. QLD temperatures are missing for 30 and 445 m elevation due to loss of data loggers. Temperatures from 445 m were replaced by adjacent stream temperatures at 482 m elevation.

Tropical (QLD) streams compared to temperate (NSW) streams had less annual temperature range (which ranged between sites from 7.2 to 11.9°C vs. 16.6 to 28.7°C, respectively) and also less diel temperature variation (7.2 to 11.9°C vs. 16.6 to 28.7°C, respectively) (Figure 2; Table S1). Relative to the temperate sites, there was less overlap of temperatures among tropical elevations (Figure 2). Tropical streams were also typically warmer with higher annual minimum (12.2 to 14.7°C vs. −0.15 to 4.5°C) and mean temperatures (17.6 to 21.5°C vs. 6.0 to 12.5°C) than temperate streams. However, annual maximum water temperatures were similar or lower in tropical than temperate streams (21.1 to 26.4°C vs. 21.1–30.3°C). The annual temperature range of tropical streams remained stable across elevations, that is, all tropical streams exhibited a similar amount of annual temperature variation (ANOVA: *F* = 0.85, *p* = .3879). Similarly, the annual temperature range in temperate streams also remained relatively stable across elevations, with only slight and not statistically significant increasing trend with elevation (ANOVA: *F* = 5.46, *p* = .1015). Because our study design resulted in the expected temperature differences in annual temperature range between the two latitudes and a declining temperature with increased elevation (Figure 2), we included consideration of the effect of the measured water temperatures at each site on *T*_br_.

**FIGURE 3:**
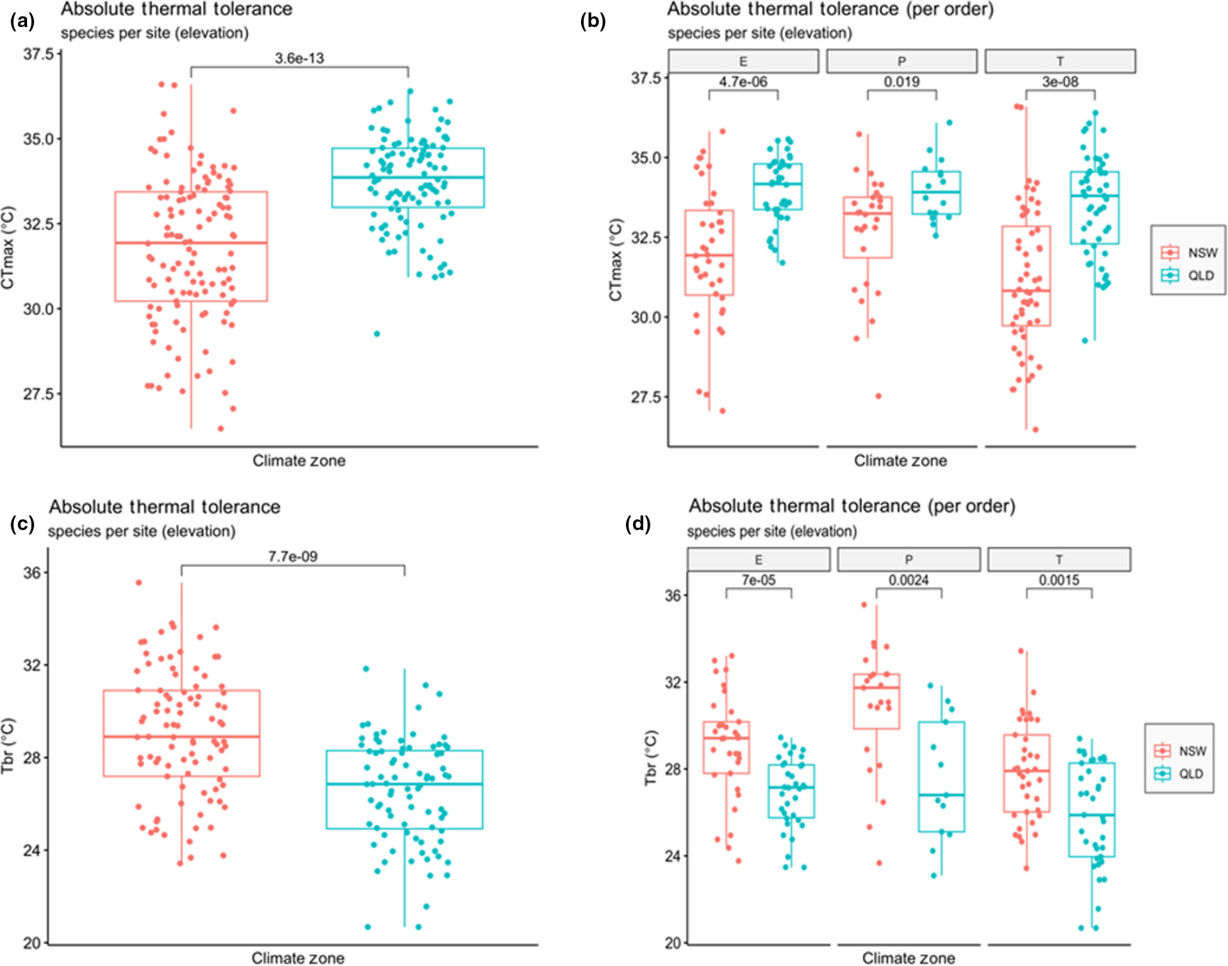
(a, b) Critical maximum temperature (CT_max_) and (c, d) thermal breadth (*T*_br_) for all test organisms between the temperate region (in NSW) and the tropical region (in QLD). Each dot represents a species at a different site (elevation). E, P and T refer to the insect orders Ephemeroptera, Plecoptera and Trichoptera, respectively.

CT_max_ values were higher in the tropical region than the temperate region (Figure 3a) (*p* < .0001), and they were broadly consistent across the three orders, that is, Ephemeroptera, Plecoptera and Trichoptera (Figure 3b). CT_min_ was also significantly lower (*p* < .0001) in temperate NSW streams than in tropical QLD streams.

**FIGURE 4:**
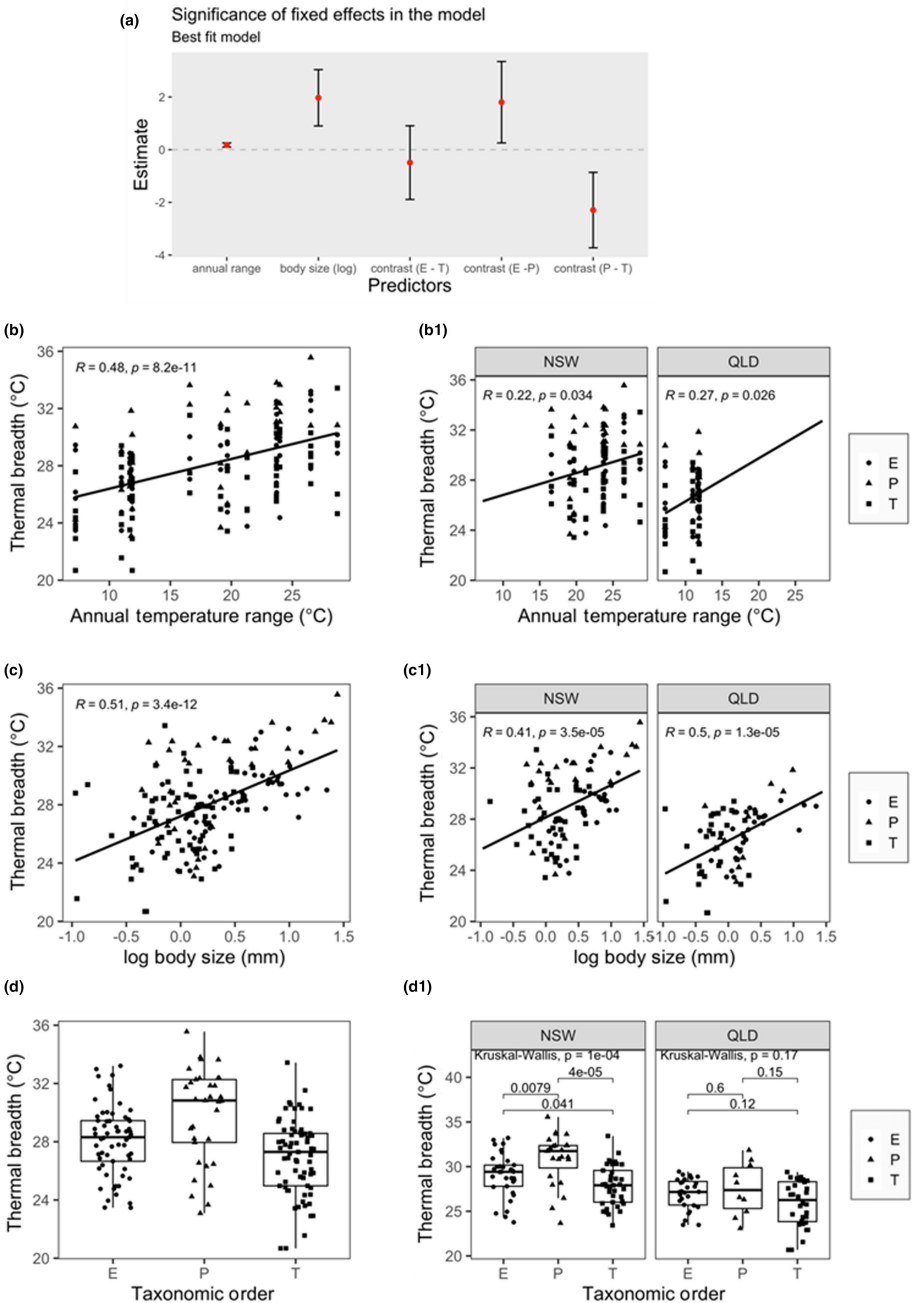
(a) Significance of fixed effects in the best‐fit model explaining thermal breadth (*T*_br_). Significant effects are annual temperature range, the body size and the taxonomic order because of plecopterans. (b, b1) Change of Thermal Breadth (*T*_br_) with increasing annual temperature range for all data and per region. (c, c1) Change of *T*_br_ with increasing body size. (d, d1) Difference of *T*_br_ between the three taxonomic orders E (Ephemeroptera), P (Plecoptera) and T (Trichoptera). Each datum point in parts b–d represent one species at a site (elevation).

As predicted by the CVH, *T*_br_ was significantly lower in tropical than temperate morphospecies (Figure 3c) with this pattern occurring across all three insect orders (Figure 3d). Also as expected, *T*_br_ significantly increased with increasing annual temperature ranges across all sites (Figure 4b) and within tropical and temperate regions (Figure 4b1).

### 3.2 Other predictors of *T*_br_

Following the predictions of the CVH, the best‐fitting mixed effects model for *T*_br_ included annual temperature range, body size (log_10_ head width) and taxonomic order (Table 1), with body size having the greatest contribution to the model (Figure 4a,c). However, as most temperature variables were highly correlated (Table S2 and Figure S1), we cannot exclude the possibility that temperature variability at diel, sampling season scales, as well as the annual mean or annual minimum temperature, contributed to the trends in *T*_br_ we observed (see Section 2). Body size was significantly higher in temperate NSW than in tropical QLD (*p* < .0366; Figure S2). *T*_br_ was higher in plecopterans than the other two orders (Figure 4d), which was mainly driven by the NSW taxa (Figure 4d1). Although some plecopterans were among the largest species we studied, many were mid‐sized (Figure 4c,c1), thus, their high *T*_br_ is not simply the result of Plecoptera being larger than the other two orders. The results of the best‐fitting model showed that the random effect ‘species’ accounts for 31%, genus for 17%, family for 0.06% and “sampling site” (elevation) for 16% of residual (Table 2). Pairwise comparison of orders using Satterthwaite approximations for degrees of freedom showed that plecopterans had significantly different *T*_br_ than trichopterans (*p* = .048) in the mixed effect model. No significant differences between ephemeropterans and plecopterans (*p* = .144) or between ephemeropterans and trichopterans (*p* = .770) were detected.

**TABLE 1:** Summary of the four best supported linear mixed models for predicting thermal breadth (*T*_br_).

Physiological trait	Fixed effects					Intercept	Df	Log Lik	AICc	∆AIC	w_i_	Conditional *r* ^2^	Marginal *r* ^2^
	Annual range	Annual maximum	Annual range * annual maximum	Body size (Log_10_)	Taxonomic order								
Thermal Breadth (*T*_br_) = CT_max_ − CT_min_	0.178			1.970	+	24.32	10	−336.02	693.50	0	0.53	.724	.473
	0.157	0.107		2.094	+	22.04	11	−335.50	694.74	1.24	0.28	.714	.468
	0.234	0.170	−0.003	2.081	+	20.53	12	−335.46	697.01	3.51	0.09	.714	.469
	0.180			1.980		24.64	9	−339.44	698.06	4.57	0.05	.712	.343

*Note*: Only the models with ΔAIC ≤5 are listed here (see Table S5 for all models). *Annual range* is the annual temperature range in °C, *Annual maximum* is the annual maximum temperature in °C, *Annual maximum:Annual range* is the interaction between the annual maximum temperature and the annual temperature range, *Body size (log)* is the log‐transformed headwidth in mm (to four decimal places). *Df* is the number of parameters in the model (default df = 2). *LogLik* (Log‐likelihood) describes how likely the model is. *AICc* is the Akaike Information Criterion for the model. ΔAIC is the difference between AICc score between the best model and the model being compared. Models with ΔAIC ≤2 have substantial support, which then lessens until with ΔAIC ≥10 no support is given to the model anymore. *W*_*i*_ (weight) is the proportion of the predictive power provided by the model. Random effects were ‘sampling site’ and the nested effect ‘family/genus/species’ (for models highlighted in grey) or ‘order/family/genus/species’ (see Table 2). The *r* ^2^ informs about the proportion of variance in *T*_br_ which is explained by the model. The conditional *r* ^2^ takes both the fixed and random effects into account. The marginal *r* ^2^ considers only the variance of the fixed effects.

**TABLE 2:** Results of the best‐fitting mixed effect model, with 163 observations, 65 species, 31 genera, 17 families and 14 sampling sites. Random effects were ‘sampling site’ and the nested effect ‘family/genus/species’. See Table S6 for these results for the second‐best model.

Random effects	Variance	Standard deviation
Species:(genus:family)	1.046	1.023
Genus:family	0.474	0.689
Family	0.152	0.389
Sampling site	0.431	0.656
Residual	2.319	1.523

Fixed effects	Estimate	Standard error	*t*‐Value
Intercept	24.317	0.871	27.933
Annual range	0.178	0.039	4.513
Body size (log)	1.970	0.534	3.688
Contrast (E‐P)	1.802	0.773	2.332
Contrast (E‐T)	−0.491	0.699	−0.702
Contrast (P‐T)	−2.292	0.716	−3.200

The second‐best model was not significantly different from the first (Table 1) and included the annual maximum temperature in addition to the variables in the best model. However, this second model showed that the annual maximum temperature has no statistically significant impact on *T*_br_ (Figure 4a; Figure S3, see also Tables S5 and S6); therefore, this model also supports the CVH. Moreover, there was a positive relationship between *T*_br_ and annual maximum temperature (Figure S3b).

Two‐way ANOVA for mean CT_min_ found values were significantly lower in NSW than QLD (*F*_1,160_ = 328, *p* < .001) and differed significantly between orders (*F*_2,160_ = 8.1, *p* < .001) but found no significant interaction between these factors (*F*_2,160_ = 328, *p* = .56). Plecoptera tended to have lower CT_min_ values (mean in NSW of 1.8°C, QLD 6.4°C) than Trichoptera (mean in NSW of 3.3°C, QLD 7.4°C) and Ephemeroptera (mean in NSW of 2.8°C, QLD 7.2°C). Ephemeroptera also tended to have lower CT_min_ values than Trichoptera.

Mean CT_max_ was significantly higher in QLD than NSW (*F*_1,195_ = 41, *p* < .001), and differed between orders (*F*_2,195_ = 3.4, *p* = .037) but there was no significant interaction between these factors (*F*_2,195_ = 1.3, *p* = .26). Plecoptera (mean in NSW of 32.7°C, QLD 33.9°C) had higher CT_max_ values than Trichoptera (mean in NSW of 31.1°C, QLD 33.7°C) but not Ephemeroptera (mean in NSW of 31.9°C, QLD 34.2°C). Ephemeroptera tended to have higher CT_max_ values than Trichoptera.

### 3.3 Intra‐ and interspecific variation in *T*_br_

**FIGURE 5:**
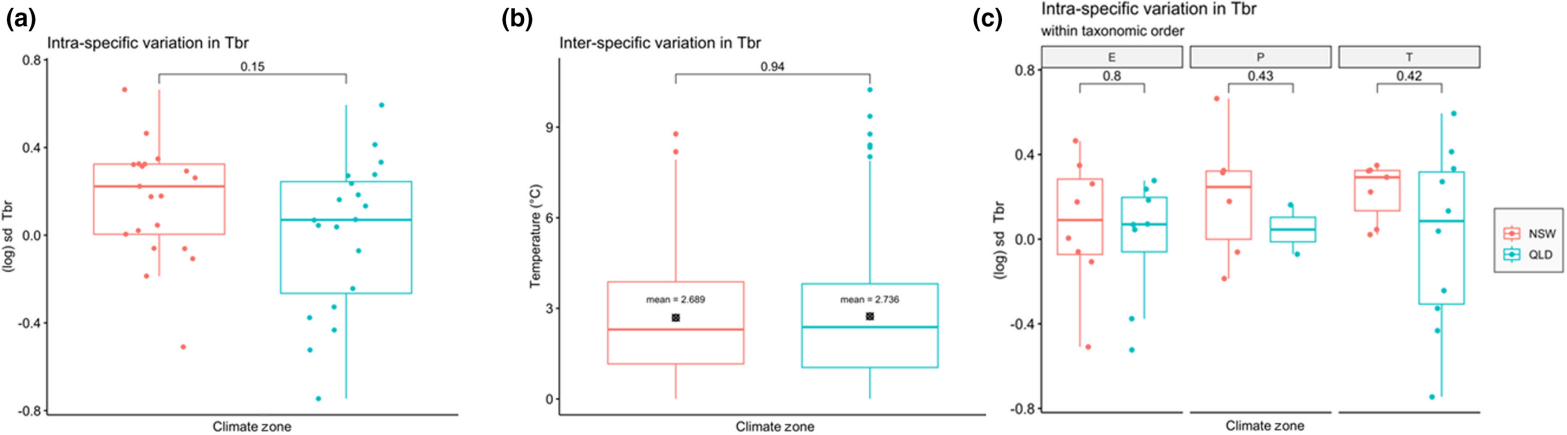
(a) Intra‐specific variation in thermal breadth (*T*_br_), (b) inter‐specific variation in *T*_br_ in the temperate location (in NSW) and the tropical location (in QLD) and (c) intra‐specific variation in *T*_br_ across orders and climate zones for each of the orders, E = Ephemeroptera, P = Plecoptera and T = Trichoptera. Each datum point refers to one species found at ≥2 sites (i.e. elevations) per climate zone.

*T*_br_ showed no difference in either intra‐ or inter‐specific variation between the temperate and tropical regions (Figure 5).

### 3.4 Testing the climate extreme hypothesis

**FIGURE 6:**
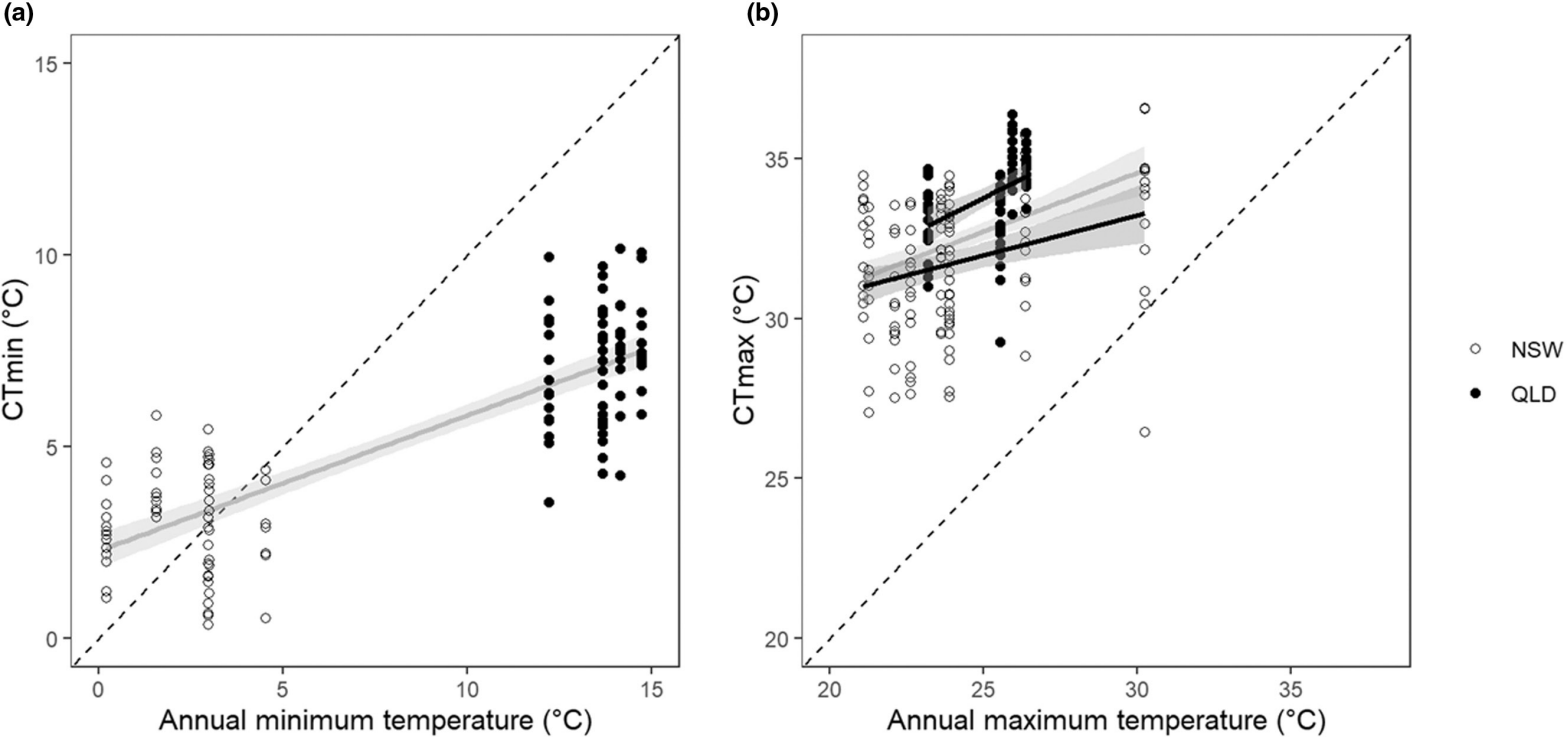
Relationships between critical thermal (CT) limits and annual extreme water temperatures (°C). Relationship between (a) CT_min_ and absolute annual minimum water temperature and (b) CT_max_ and absolute annual maximum water temperatures. Data points indicate a morphological species at a site in NSW (temperate climate, open circles) or QLD (tropical climate, solid circles). Dashed lines are the 1:1 relationship, that is, showing where CT_min_ or CT_max_ equals the annual minimum or maximum, respectively, water temperature. Solid lines are the ordinary least squares regression lines and shading indicates their 95% confidence intervals. Solid grey lines represent these regressions using all data, while those in black represent statistically significant regressions using data for either NSW or QLD.

Across both climatic regions, absolute minimum (*F*_1,164_ = 365, *p* < .001) and maximum annual (*F*_1,193_ = 142, *p* < .001) annual water temperatures were highly correlated with CT_min_ and CT_max_, respectively (Figure 6). While more variability was explained by the regression model for CT_min_ (adjusted *r* ^2^ = .69) than CT_max_ (adjusted *r* ^2^ = .15) both regression lines had a similar positive slope (CT_min_: 0.35 ± 0.018, CT_max_: 0.37 ± 0.062). Separate regressions for each climatic region found no significant relationship between minimum water temperature and CT_min_ (Temperate: *F*_1,104_ = 2.26, *p* = .14, adjusted *r* ^2^ = .012; Tropical: *F*_1,58_ = 1.75, *p* = .093, adjusted *r* ^2^ = .032). CT_min_ values were warmer than the annual minimum observed temperature at their collection site from some species in temperature regions, but all species in the tropical regions had cooler CT_min_ values than the minimum temperature at their collection site (Figure 6a). Separate regressions found significant relationships between maximum water temperature and CT_max_ in both regions (Temperate: *F*_1,122_ = 54.9, *p* < .001, adjusted *r* ^2^ = .087; Tropical: *F*_1,69_ = 15.5, *p* < .001, adjusted *r* ^2^ = .17), although the slopes were different (Temperate: 0.25 ± 0.07, Tropical 0.49 ± 0.12). In both climatic regions, CT_max_ values were greater than the absolute maximum annual temperature for all species except one.

## 4 DISCUSSION

The laboratory measured thermal tolerances of freshwater insects from the orders Ephemeroptera (mayflies), Plecoptera (stoneflies) and Trichoptera (caddisflies), from temperate and tropical eastern Australia, support the predictions of the CVH (Janzen, 1967). Thermal breadth (*T*_br_) was generally greater at our temperate than at our tropical location (Figure 2) because of lower CT_min_ values at our temperature location relative to our tropical location (Figure 6). *T*_br_ tended to increase with increasing annual temperature ranges across all sites and in both temperate and tropical locations (Figure 4b,b1). A meta‐analysis (Sunday et al., 2011) observed that the CVH was better supported in the Northern Hemisphere than in the Southern Hemisphere. Yet our results from the Southern Hemisphere corroborate the findings of Shah, Gill, et al. (2017) who tested the CVH in phylogenetically paired aquatic insects in Colorado, USA and in Ecuador. Indeed, support for the CVH extends beyond aquatic insects to other freshwater (Gutiérrez‐Pesquera et al., 2016; Markle & Kozak, 2018), marine, and terrestrial (Sunday et al., 2011) ectotherms. However, *T*_br_ correlates less tightly with temperature variation in terrestrial insects (Kellermann et al., 2012; Kimura, 2004; Nowrouzi et al., 2018). The aforementioned meta‐analysis also found the rate of increase in *T*_br_ with increasing latitude was lower in marine than terrestrial species (Sunday et al., 2011). In aquatic habitats water tends to generate more spatially homogeneous thermal conditions than in most terrestrial habitats, thus there is less potential for behavioural thermoregulation in aquatic relative to terrestrial habitats (Havird et al., 2020; Muñoz & Bodensteiner, 2019; Shah et al., 2020; Woods et al., 2015). The apparent difference between terrestrial insects (Kellermann et al., 2012, Kimura, 2004, Nowrouzi et al., 2018) in contrast to the current and other freshwater studies (Gutiérrez‐Pesquera et al., 2016; Markle & Kozak, 2018; Shah, Gill, et al., 2017) and marine studies (Sunday et al., 2011) suggests differences between the CVH's applicability in freshwater and marine habitats.

Our results do not support the hypotheses formulated by Payne and Smith (2017), and other similar hypotheses (Dillon et al., 2010; Gillooly et al., 2006), which posit that high temperatures constrain ectotherms ability to tolerate high annual thermal variability. Payne and Smith (2017) predicted that high temperatures would reduce *T*_br_ (Payne & Smith, 2017). Yet, our best model of *T*_br_ found no evidence that the annual maximum temperature affected *T*_br_. While our second‐best model included annual maximum temperature, *T*_br_ tended to increase with increasing annual maximum temperature (Figure S3b), that is, annual maximum temperature had the opposite effect on *T*_br_ to Payne and Smith's (2017) prediction. However, our results cannot exclude the possibility that high temperatures may constrain an organism's ability to tolerate short term (e.g. acute and diel) thermal variability (Kefford et al., 2022), and further studies are needed to resolve such unknowns.

Consistent with the CEH (Pither, 2003; Sunday et al., 2019) using data from both climatic zones CT_min_ and CT_max_ were positively related to minimum and maximum water temperatures, respectively (Figure 6). Sunday et al. (2019) observed similar relationships with predicted air temperature for freshwater and terrestrial ectotherms. The relationship between CT_min_ and minimum water temperature in the current study was driven by differences between the temperate and tropical locations, with no apparent effect of elevation. Moreover, in the temperate zone, some species had CT_min_ greater than the minimum temperature recorded at their collection site. For example, species at sites with a minimum temperature of about 0°C have CT_min_ values up to almost 5°C. This apparent contradiction implies non‐viable local population sustained by migration from elsewhere, seasonal elevational migration or seasonal lowering of CT_min_. Seasonal lowering of CT_min_ could be due to either life‐stage specific changes in CT_min_ which have been observed in Ephemeroptera (Uno & Stillman, 2020) or acclimation (Fangue & Bennett, 2003; Havird et al., 2020). In contrast to CT_min_, CT_max_ values were related to differences in annual extreme temperatures between the climatic zones and annual mean temperatures along elevational gradients. In summary, thermal extremes can explain variation in maximum thermal tolerance better than minimum thermal tolerance.

Annual temperature range was highly correlated with multiple other aspects of thermal regimes (Table S2, Figure S5), including with diel temperature variability as Shah, Gill, et al. (2017) also observed. It is impossible to disentangle the influence of these individual (but corelated) aspects of thermal regimes on *T*_br_ and other components of thermal biology. Parts of the world do have highly contrasting diel and annual thermal variability (Geerts, 2002, 2003; Wang & Dillon, 2014). For instance, many humid high‐latitude locations have minimal diel variability but high annual variability (Wang & Dillon, 2014). In contrast, tropical high‐elevational mountains can have high diel variability but limited annual variability (Mani, 1968). There is an urgent need to consider the influence of various aspects of thermal regimes on *T*_br_ at a wide range of locations with contrasting aspects in thermal climate to disentangle the effects of multiple aspects of thermal regimes (Kefford et al., 2022).

Larger individuals tended to have wider *T*_br_ than smaller individuals (Figure 4c). Small ectotherms generally have lower CT_min_ values (Leiva et al., 2019) and higher CT_max_ values (Leiva et al., 2019; Peralta‐Maraver & Rezende, 2021) than larger ectotherms. However, this relationship is also altered by genome size and differs between air and water‐breathing ectotherms (Leiva et al., 2019). For water‐breathing ectotherms (such as the freshwater insects we studied) the reduced CT_max_ values of larger individuals are only apparent with long‐term thermal tolerance trials (Leiva et al., 2019). Our measurements of CT_max_ involved brief exposures (temperature was increased at 0.25°C/min with the longest experiment lasting about 85 min). The ability of these measurements of short‐term upper thermal tolerance to predict long‐term upper thermal tolerance remains uncertain (Ørsted et al., 2022; Rezende et al., 2014).

Size was also a confounding variable in comparing temperate and tropical locations. Individuals were generally larger in our temperate compared to our tropical location (Figure S2). This relationship could be a consequence of cooler temperatures at the temperate location (Figure 2) leading to phenotypic plasticity, as described by the Temperature‐Size Rule (Kingsolver & Huey, 2008) and documented in Ephemeroptera (Atkinson, 1994; Sweeney et al., 2018). Additionally, natural selection might cause populations and species to be larger in the cooler temperate location than the warmer tropical location, consistent with Bergmann's Rule (although with many counter‐examples, Kingsolver & Huey, 2008; Papandreou et al., 2023). Thus, whether a large size in the temperate location is a cause of wide *T*_br_ or an effect of climate that may then alter thermal tolerance in an exposure‐duration‐dependent manner (Peralta‐Maraver & Rezende, 2021), will require further investigation.

We observed that Plecoptera tended to have wider *T*_br_ compared to Ephemeroptera and Trichoptera because Plecoptera tended to have both lower CT_min_ and higher CT_max_ values than the other orders. The wide *T*_br_ for plecopterans compared to ephemeropterans and trichopterans, we and Shah, Gill, et al. (2017) observed contrasts with the general expectation that Plecoptera are restricted to cooler waters, with a presumed narrow tolerance range and high vulnerability to climate change (Fochetti & De Figueroa, 2008). Indeed, plecopterans were assessed as the most vulnerable group in global warming scenarios in mountainous running water ecosystems (Besacier Monbertrand et al., 2019). One explanation for wide thermal tolerance in plecopterans compared to the other two orders is that plecopterans evolved in temperate environments generating wide *T*_br_ (Polato et al., 2018), which persisted after moving into tropical locations (Bennett et al., 2021; Wake et al., 1983). However, plecopterans are among the oldest extant groups of insects, originating in the Carboniferous approximately 300–350 million years ago (Cui et al., 2016). CT_max_ and CT_min_ have been estimated to evolve in ectotherms at 0.784 ± 0.225°C per mya^−1^ and 1.22 ± 0.385°C per mya^−1^, respectively (Bennett et al., 2021), providing ample time for *T*_br_ to adapt to tropical climate conditions, even if they moved there in comparatively recent earth history. Overall, our results imply that regardless of the climate zone, plecopterans may have comparatively higher ability to cope physiologically with short‐term thermal stress than the other two orders. The degree to which this short‐term tolerance extends to tolerance of longer‐term temperature change remains to be investigated (Shah et al., 2023).

The inter‐specific and intra‐specific variation in *T*_br_ was similar in both the temperate and tropical streams. Nati et al. (2021) observed that tropical freshwater fish tended to have less intra‐specific variation in CT_max_ than temperate freshwater fish, although this trend was greater in the Northern than in the Southern Hemisphere. We hypothesised that the similarity of annual temperature range we observed between sites within climate zones (Figure 2) may have contributed to the similarity of intra‐ and inter‐specific in *T*_br_ between the climate zones. That is, regardless of elevation, species experienced similar annual temperature variation within temperate or tropical stream, so plastic responses such as acclimation (Havird et al., 2020), epigenetic changes, genetic drift and adaptive evolution (Hoffmann et al., 2013) within the climate zone tended to result in species having similar amounts of intra‐species variation in *T*_br_. Likewise, the similarity of annual temperature range within climate zone would tend not to be selected for speciation and population‐level effects across the species' range (Slatyer et al., 2016) leading to similar inter‐specific variation between the climate zones. The similarity of inter‐specific and intra‐specific variation in *T*_br_ across climate zones implies a similar effect on the vulnerability of species and community functions, respectively, to changes in annual scale temperature variability (Franken et al., 2018; Herrando‐Pérez et al., 2019; Nati et al., 2021).

In conclusion, we find strong support for the Climate Variability Hypothesis (Janzen, 1967) and no support for the hypothesis that high maximum temperatures on an annual scale constrain individuals' ability to tolerate a wide range of temperatures (Dillon et al., 2010; Gillooly et al., 2006; Payne & Smith, 2017). We were unable to investigate the potential influence of aspects of thermal regimes on thermal breadth other than the annual temperature range and annual maximum temperature (e.g. diel temperature cycling and minimum temperature) due to the highly intercorrelated nature of the data. Therefore, we cannot exclude the possibility that aspects of thermal regimes other than annual temperature range (partly) influenced *T*_br_. We do also find support for the CEH (Pither, 2003; Sunday et al., 2019) but more support for this hypothesis in explaining CT_max_ than CT_min_. We suggest that the CVH and the CEH should not be couched in terms of alternative hypotheses as they both predict different aspects of thermal biology. The CVH predicts traits associated with living in a wider or narrower range of temperatures, while the CEH predicts thermal tolerance traits associated with living in extreme temperatures. There is a pressing need to consider the singular and cumulative effect of a wide range of aspects of thermal regimes on organismal response to climate warming (Kefford et al., 2022). In addition to climate variables, *T*_br_ is associated with biological variables, such as size and taxonomic identity, although whether size influences *T*_br_ or is itself a consequence of the thermal regime in which an organism lives is unclear.

## AUTHOR CONTRIBUTIONS

**Beatrice S. Dewenter:** Conceptualization (equal); data curation (lead); formal analysis (lead); investigation (lead); methodology (supporting); visualization (lead); writing – original draft (lead). **Alisha A. Shah:** Conceptualization (equal); formal analysis (supporting); methodology (lead); writing – review and editing (supporting). **Jane Hughes:** Conceptualization (equal); funding acquisition (equal); supervision (supporting); writing – review and editing (supporting). **N. LeRoy Poff:** Conceptualization (equal); funding acquisition (supporting); methodology (supporting); supervision (supporting); writing – review and editing (supporting). **Ross Thompson:** Conceptualization (equal); funding acquisition (supporting); investigation (supporting); methodology (supporting); project administration (supporting); resources (supporting); supervision (supporting); writing – review and editing (supporting). **Ben J. Kefford:** Conceptualization (equal); data curation (supporting); formal analysis (supporting); funding acquisition (lead); investigation (supporting); methodology (supporting); project administration (lead); resources (equal); supervision (lead); visualization (supporting); writing – review and editing (lead).

## CONFLICT OF INTEREST STATEMENT

The authors declare that there are no conflicts of interest pertaining to this research.

### OPEN RESEARCH BADGES

Data has been uploaded to Dryad: https://doi.org/10.5061/dryad.9cnp5hqs1.

## Supporting information


Data S1.

## Data Availability

The data that support the findings of this study are available in Supplementary Tables. The complete primary data on which this paper is based upon will be made publicly available 1 year after the publication of this paper.
