# Supplementary material for: The thermal breadth of temperate and tropical freshwater insects supports the climate variability hypothesis
Source: Ecol Evol. 2024 Feb 23;14(2):e10937. doi: 10.1002/ece3.10937 (PMC10891360; doi:10.1002/ece3.10937)
Supplement: Supplementary file 1 — Data S1. [file ECE3-14-e10937-s001.docx]

Supplementary material for: **The thermal breadth of temperate and tropical freshwater insects supports the climate variability hypothesis**

Beatrice S. Dewenter^1#^; Alisha A. Shah^2^ ([aashah@msu.edu](mailto:aashah@msu.edu)); Jane Hughes^4^ [jane.hughes@griffith.edu.au](mailto:jane.hughes@griffith.edu.au)); N. LeRoy Poff^1,3^ ([LeRoy.Poff@canberra.edu.au](mailto:LeRoy.Poff@canberra.edu.au)); Ross Thompson^1^ ([ross.thompson@canberra.edu.au](mailto:ross.thompson@canberra.edu.au)); Ben J. Kefford^1^ ([ben.kefford@canberra.edu.au](mailto:ben.kefford@canberra.edu.au))

Corresponding Author: Ben J. Kefford, ORCID: 0000-0001-6789-4254

# After writing several drafts of this paper, Beatrice Dewenter died unexpectedly from a medical condition.

**Affiliations:**^1^ Centre for Applied Water Science, Institute for Applied Ecology, University of Canberra, ACT, Australia
^2^ W.K. Kellogg Biological Station, Department of Integrative Biology, Michigan State University, MI, USA
^3^ Department of Biology, Colorado State University, CO, USA
^4^ School of Environment and Science, Griffith University, QLD, Australia

Supplementary Table S1. Collection sites information with temperature, water quality and habitat data. The water qulity and habitat data relate to that measured durinring collection of the insects for CT_min_ and CT_max_ measurments. Sn = Snowy River, Mu = Murray River, Ba = Barron River, Au = autumn (March to May), Dr = dry season (July to August).

| **Sample Sites** | ***Climate*** | Temperate (NSW) | | | | | | | | | Tropical (QLD) | | | | | |
| --- | --- | --- | --- | --- | --- | --- | --- | --- | --- | --- | --- | --- | --- | --- | --- | --- |
|  | ***Stream*** | Jacobs River | Pheasants Creek | Yellow Bog Creek | Khancoban Creek | Mowambah River | Ogilvies Creek | Dicky Cooper Creek | Munyang River | Snowy River | Stoney Creek | Dowah Creek | Streets Creek | Shoteel Creek | Breach Creek | Emerald Creek |
|  | ***Catchment*** | Sn | Mu | Mu | Mu | Sn | Mu | Mu | Sn | Sn | Ba | Ba | Ba | Ba | Ba | Ba |
|  | ***Latitude*** | -36.7501 | -36.3646 | -36.01455 | -36.1717 | -36.5012 | -36.049 | -36.25433 | -36.30017 | -36.45779 | -16.87433 | -16.96333 | -16.8264 | -16.96072 | -17.11601 | -17.07892 |
|  | ***Longitude*** | 148.4473 | 148.1655 | 148.20985 | 148.1762 | 148.4814 | 148.3298 | 148.36081 | 148.38753 | 148.29447 | 145.67025 | 145.67944 | 145.65449 | 145.64617 | 145.62919 | 145.62053 |
|  | ***Elevation (m)*** | 278 | 415 | 662 | 796 | 1140 | 1341 | 1500 | 1710 | 1943 | 30 | 97 | 364 | 445* | 705 | 951 |
|  | ***Barometric Pressure (mm Hg)*** | 980 | 971 | 943 | NA | 879 | 867 | 839 | 827 | 806.2 | 1009 | 998 | 970 | 958 | 934 | 908 |
| **365 days** | ***Annual minimum (°C)*** | 1.57 | 4.5 | 2.99 | 3 | 0.2 | -0.01 | -0.1 | -0.08 | -0.15 | n.a. | 15 | 14 | 13.67 | 12 | 14 |
|  | ***Annual maximum (°C)*** | 30.3 | 21 | 22.1 | 23 | 23.89 | 21.28 | 23.6 | 23.9 | 26.36 | n.a. | 26 | 26 | 25.54 | 23 | 21 |
|  | ***Annual temperarure range(°C)*** | 28.7 | 17 | 19.1 | 20 | 23.69 | 21.29 | 23.7 | 24 | 26.51 | n.a. | 12 | 12 | 11.87 | 11 | 7.2 |
|  | ***Annual mean (°C)*** | 12.5 | 11 | 10.3 | 9.8 | 8.79 | 8.01 | 6.93 | 6.1 | 6.02 | n.a. | 21 | 21 | 20.29 | 18 | 18 |
|  | **sd** | 6.23 | 3.3 | 4.41 | 4.4 | 5.32 | 4.12 | 5.12 | 5.01 | 6.66 | n.a. | 2.6 | 2.3 | 2.37 | 2.2 | 1.8 |
|  | ***Mean diel variability per year (°C)*** | 3.33 | 2.4 | 2.2 | 2.8 | 2.27 | 4.32 | 4.17 | 3.59 | 3.7 | n.a. | 1 | 1.2 | 0.98 | 1.2 | 0.6 |
|  | **sd** | 2.05 | 1.4 | 1.29 | 1.8 | 1.38 | 2.52 | 2.51 | 2.79 | 3.61 | n.a. | 0.4 | 0.8 | 0.45 | 0.6 | 0.3 |
| **Sampling season** | ***Season*** | Au | Au | Au | Au | Au | Au | Au | Au | Au | Dr | Dr | Dr | Dr | Dr | Dr |
|  | ***Seasonal temperature range (°C)*** | 19.2 | 11 | 12.6 | 13 | 14.23 | 15.17 | 17.2 | 16.9 | 18.19 | n.a. | 7.8 | 8.7 | 7.73 | 7.2 | 4 |
| **Water Quality** | ***Alkalinity (mg/L)*** | 10 | 11 | 10 | 12 | 12 | 5 | 6 | 8 | 5 | 12 | 11 | 10 | 12 | 8 | 6 |
|  | ***Nitrite (mg/L)*** | 0.02 | 0 | 0 | 0 | 0 | 0 | 0.02 | 0 | 0 | 0 | 0 | 0 | 0 | 0 | 0 |
|  | ***Thotal Phosphorus (mg/L)*** | 0 | 0 | 0 | 0 | 0 | 0 | 0 | 0 | 0 | 0 | 0 | 0 | 0 | 0 | 0 |
|  | ***Nitrogen Ammonia (mg/L)*** | 0.09 | -0.1 | 0 | 0 | 0.07 | -0.03 | 0.27 | 0.06 | 0.01 | 0 | 0 | 0 | 0.02 | 0 | 0.1 |
|  | ***Nitrate (mg/L)*** | 0.3 | 0.2 | 0.2 | 0.2 | 0.3 | 0.1 | 0.3 | 0.4 | 0.2 | -0.2 | 0.3 | 0 | 0.2 | 0.3 | 0.2 |
|  | ***Conductivity (mS/cm)*** | 0 | 0 | 0 | 0 | 0.003 | 0 | 0.01 | 0.01 | 0.007 | 0.049 | 0 | 0 | 0.047 | 0 | 0 |
|  | ***pH*** | 7.49 | 7.4 | 7.46 | 6.7 | 6.75 | 7.12 | 7.19 | 6.58 | 7.19 | 7.07 | 6.6 | 6.4 | 7.16 | 7.4 | 6.3 |
|  | ***Dissolved Oxygen (% sat)*** | 83.4 | 76 | 94 | 77 | 78.8 | 70.8 | 82.1 | 73.7 | 78 | 73.4 | 86 | 72 | 79.6 | 77 | 77 |
|  | ***Turbidity (NTU)*** | 0.8 | 0.9 | 1.6 | 0.2 | 0 | 1.4 | 0 | 0 | 0 | 0 | 2.3 | 0 | 0 | 0 | 0 |
| **Habitat** | ***Velocity (m/s on bottom of stream)*** | 0.23 | 0.3 | 0.48 | 0.3 | 0.35 | 0.24 | 0.24 | 0.27 | 0.33 | 0.6 | 0.8 | 0.4 | 0.18 | 0.4 | 0.5 |
|  | ***Stream depth (cm)*** | 29.7 | 12 | 18.3 | 14 | 20.7 | 12.5 | 16 | 13.4 | 5.7 | 15.7 | 18 | 10 | 8.1 | 14 | 20 |

* Temperature data at the 445m QID site came from nearby site with an elervation of 482m

Supplementary Table S2. Correlation matrix of Pearson’s r values for the six explanatory temperature variables of the PCA (Supplementary Figure S5), not log-transformed. Abbreviations are defined in Supplementary Table S5. Correlations >= 0.7 threshold are highlighted in grey.

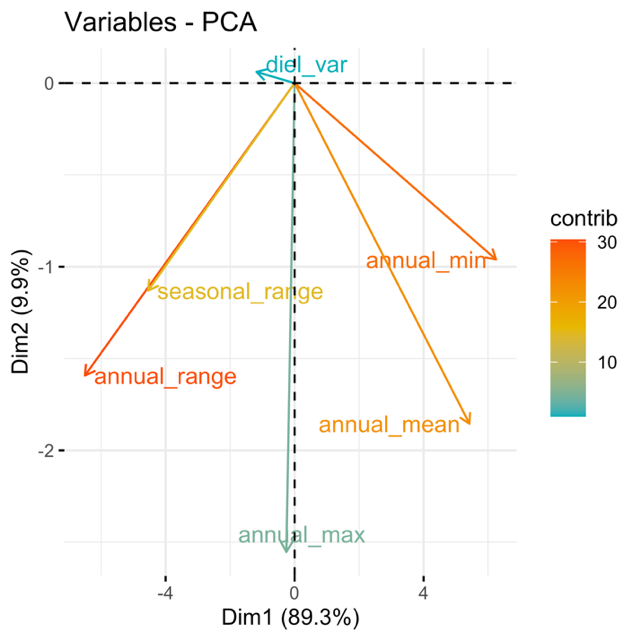


Supplementary Figure S1. Principal coordinate analysis (PCA) of the seven explanatory temperature variables in Supplementary Table S2 (see Supplementary Table S5 for abbreviations and data). Dimension 1 (x-axis) explained 69.6 %, and Dimension 2 (y-axis) explained 21.8 % of the variation among the variables. Colours indicate each variable’s contribution (contrib) to the variation explained in the first two dimension.

Supplementary Table S3. Critical thermal minimum (CT_min_), critical thermal maximum (CT_max_) and/or CT_min_ and/or thermal breadth (T_b_) (all in °C) for each morphospecies at each site (elevation) with ≥ 3 individuals measured. Where E = Ephemeroptera (mayfliies), T = Trichoptera (caddisflies), P = Plecoptera (stoneflies), n = number of replicate individual of the taxa indicated at that site, sd=standard deviation, hw = head width (mm) TB=T_b_ or thermal breadth (°C), NA= no data. So, for example, n CTmin = the number of replicate individuals that CTmin was measured for the indicated taxa at the indicates site (elevation) and TB mean hw sd = the standard deviation of the head width (mm) of those individuals from which mean T_b_ was estimated for the indicated taxa and indicate site (elevation).

| Location | Climate | Elevation (m) of site | Order | Family | Genus | Species | n CTmin | CTmin mean | CTmin sd | CTmin mean hw | CTmin sd hw | n CTmax | CTmax mean | CTmax sd | CTmax mean hw | CTmax sd hw | n TB | TB | TB sd | TB mean hw | TB mean hw sd |
| --- | --- | --- | --- | --- | --- | --- | --- | --- | --- | --- | --- | --- | --- | --- | --- | --- | --- | --- | --- | --- | --- |
| NSW | Temperate | 278 | E | Ameletopsidae | Mirawara | Mirawara sp sp | 3 | 3.37 | 0.06 | 2.3 | 0.14 | NA | NA | NA | NA | NA | NA | NA | NA | NA | NA |
| NSW | Temperate | 278 | E | Baetidae | Offadens | Offadens o hickmani complex | 9 | 3.58 | 0.72 | 0.9 | 0.08 | 8 | 32.98 | 4.66 | 0.79 | 0.11 | 17 | 29.4 | 5.38 | 0.845 | 0.19 |
| NSW | Temperate | 278 | E | Coloburiscidae | Coloburiscoides | Coloburiscoides sp | 9 | 3.7 | 1.49 | 1.75 | 0.28 | 9 | 33.87 | 0.69 | 1.86 | 0.23 | 18 | 30.17 | 2.18 | 1.805 | 0.51 |
| NSW | Temperate | 278 | E | Leptophlebiidae | Atalophlebia | Atalophlebia sp AV2 | NA | NA | NA | NA | NA | 4 | 34.73 | 5.29 | 1.83 | 0.13 | NA | NA | NA | NA | NA |
| NSW | Temperate | 278 | E | Leptophlebiidae | Atalophlebia | Atalophlebia sp AV4 | 3 | 4.33 | 1.36 | 1.22 | 0.09 | NA | NA | NA | NA | NA | NA | NA | NA | NA | NA |
| NSW | Temperate | 278 | E | Leptophlebiidae | Austrophlebioides | Austrophlebioides pusillus | 9 | 3.3 | 0.5 | 1.72 | 0.16 | 9 | 32.18 | 3.6 | 1.79 | 0.12 | 18 | 28.88 | 4.1 | 1.755 | 0.28 |
| NSW | Temperate | 278 | E | Leptophlebiidae | Nousia | Nousia sp AV1 | NA | NA | NA | NA | NA | 3 | 34.7 | 1.67 | 1.33 | 0.05 | NA | NA | NA | NA | NA |
| NSW | Temperate | 278 | P | Gripopterygidae | Riekoperla | Riekoperla rugosa group | 17 | 3.79 | 2.12 | 1.09 | 0.07 | 9 | 34.62 | 0.5 | 1.07 | 0.08 | 26 | 30.83 | 2.62 | 1.08 | 0.15 |
| NSW | Temperate | 278 | T | Calamoceratidae | Anisocentropus | Anisocentropus sp | NA | NA | NA | NA | NA | 6 | 34.07 | 2.98 | 0.79 | 0.11 | NA | NA | NA | NA | NA |
| NSW | Temperate | 278 | T | Calocidae | Tamasia | Tamasia sp AV1 | 7 | 4.84 | 1.34 | 0.63 | 0.03 | 7 | 30.86 | 2.44 | 0.65 | 0.03 | 14 | 26.02 | 3.78 | 0.64 | 0.06 |
| NSW | Temperate | 278 | T | Hydrobiosidae | Taschorema complex | Taschorema evansi | NA | NA | NA | NA | NA | 3 | 30.87 | 0.32 | 1.09 | 0.23 | NA | NA | NA | NA | NA |
| NSW | Temperate | 278 | T | Hydrobiosidae | Ulmerochorema | Ulmerochorema rubiconum | 9 | 5.82 | 0.54 | 1.08 | 0.18 | 4 | 30.47 | 1.66 | 1.24 | 0.05 | 13 | 24.65 | 2.2 | 1.16 | 0.23 |
| NSW | Temperate | 278 | T | Hydropsychidae | Cheumatopsyche | Cheumatopsyche sp AV4 | NA | NA | NA | NA | NA | 3 | 36.57 | 0.55 | 0.95 | 0.2 | NA | NA | NA | NA | NA |
| NSW | Temperate | 278 | T | Leptoceridae | Triplectides | Triplectides ciuskus ciuskus | 9 | 3.17 | 1.28 | 0.81 | 0.31 | 9 | 36.6 | 3.69 | 0.92 | 0.23 | 18 | 33.43 | 4.97 | 0.865 | 0.54 |
| NSW | Temperate | 278 | T | Philopotamidae | Chimarra | Chimarra monticola | 8 | 4.7 | 0.21 | 0.89 | 0.03 | 8 | 34.27 | 1.6 | 0.89 | 0.02 | 16 | 29.57 | 1.81 | 0.89 | 0.05 |
| NSW | Temperate | 278 | T | Philorheithridae | Kosrheithrus | Kosrheithrus tillyardi | NA | NA | NA | NA | NA | 3 | 26.47 | 0.75 | 0.77 | 0.62 | NA | NA | NA | NA | NA |
| NSW | Temperate | 415 | E | Baetidae | Offadens | Offadens o hickmani complex | 9 | 3 | 0.71 | 1.04 | 0.07 | 16 | 30.06 | 1.84 | 1.06 | 0.08 | 25 | 27.06 | 2.55 | 1.05 | 0.15 |
| NSW | Temperate | 415 | E | Coloburiscidae | Coloburiscoides | Coloburiscoides sp | 9 | 2.89 | 0.62 | 1.99 | 0.26 | 9 | 32.92 | 0.79 | 1.96 | 0.31 | 18 | 30.03 | 1.41 | 1.975 | 0.57 |
| NSW | Temperate | 415 | E | Leptophlebiidae | Nousia | Nousia sp AV1 | NA | NA | NA | NA | NA | 3 | 31.03 | 0.23 | 1.79 | 0.16 | NA | NA | NA | NA | NA |
| NSW | Temperate | 415 | E | Leptophlebiidae | Nousia | Nousia spp AV11 | 11 | 2.22 | 0.34 | 1.78 | 0.21 | 10 | 30.72 | 0.7 | 1.77 | 0.22 | 21 | 28.5 | 1.04 | 1.775 | 0.43 |
| NSW | Temperate | 415 | P | Eustheniidae | Cosmioperla | Cosmioperla kuna | 7 | 0.54 | 0.39 | 2.3 | 0.69 | 8 | 34.17 | 0.74 | 2.34 | 0.73 | 15 | 33.63 | 1.13 | 2.32 | 1.42 |
| NSW | Temperate | 415 | P | Gripopterygidae | Dinotoperla | Dinotoperla eucumbene | 9 | 2.23 | 0.71 | 0.73 | 0.07 | 9 | 34.5 | 0.35 | 0.76 | 0.03 | 18 | 32.27 | 1.06 | 0.745 | 0.1 |
| NSW | Temperate | 415 | P | Gripopterygidae | Trinotoperla | Trinotoperla sp | NA | NA | NA | NA | NA | 4 | 33.75 | 1.16 | 0.75 | 0.1 | NA | NA | NA | NA | NA |
| NSW | Temperate | 415 | T | Calocidae | Tamasia | Tamasia sp AV1 | 7 | 4.13 | 0.8 | 0.73 | 0.09 | 7 | 31.63 | 1.38 | 0.8 | 0.06 | 14 | 27.5 | 2.18 | 0.765 | 0.15 |
| NSW | Temperate | 415 | T | Hydropsychidae | Cheumatopsyche | Cheumatopsyche sp AV3 | 9 | 2.17 | 0.59 | 1.02 | 0.03 | 9 | 33.7 | 0.55 | 1.04 | 0.04 | 18 | 31.53 | 1.14 | 1.03 | 0.07 |
| NSW | Temperate | 415 | T | Leptoceridae | Triplectides | Triplectides proximus | NA | NA | NA | NA | NA | 3 | 33.43 | 0.4 | 0.68 | 0.17 | NA | NA | NA | NA | NA |
| NSW | Temperate | 415 | T | Philorheithridae | Kosrheithrus | Kosrheithrus tillyardi | 3 | 4.4 | 0 | 0.75 | 0.19 | 3 | 30.5 | 3.5 | 1.05 | 0.47 | 6 | 26.1 | 3.5 | 0.9 | 0.66 |
| NSW | Temperate | 662 | E | Baetidae | Offadens | Offadens o hickmani complex | 15 | 4.66 | 1.24 | 1.13 | 0.05 | 17 | 29.61 | 1.54 | 1.16 | 0.11 | 32 | 24.95 | 2.78 | 1.145 | 0.16 |
| NSW | Temperate | 662 | E | Coloburiscidae | Coloburiscoides | Coloburiscoides sp | 9 | 2.06 | 0.33 | 2.88 | 0.25 | 9 | 32.69 | 1.1 | 2.58 | 0.53 | 18 | 30.63 | 1.43 | 2.73 | 0.78 |
| NSW | Temperate | 662 | E | Leptophlebiidae | Austrophlebioides | Austrophlebioides marchanti | 6 | 4.15 | 0.36 | 2.58 | 0.19 | 6 | 33.57 | 0.62 | 2.39 | 0.22 | 12 | 29.42 | 0.98 | 2.485 | 0.41 |
| NSW | Temperate | 662 | E | Leptophlebiidae | Austrophlebioides | Austrophlebioides marchanti sp | 3 | 3.6 | 0.17 | 2.5 | 0.09 | 3 | 31.33 | 2.8 | 1.87 | 0.24 | 6 | 27.73 | 2.97 | 2.185 | 0.33 |
| NSW | Temperate | 662 | E | Leptophlebiidae | Nousia | Nousia spp AV11 | 3 | 2.83 | 0.25 | 2.11 | 0.49 | NA | NA | NA | NA | NA | NA | NA | NA | NA | NA |
| NSW | Temperate | 662 | P | Eustheniidae | Cosmioperla | Cosmioperla kuna | 4 | 1.9 | 0.91 | 2.51 | 0.63 | 5 | 32.8 | 1.33 | 2.87 | 0.39 | 9 | 30.9 | 2.24 | 2.69 | 1.02 |
| NSW | Temperate | 662 | P | Gripopterygidae | Dinotoperla | Dinotoperla brevipennis | 3 | 4.03 | 0.85 | 1.35 | 0.16 | 3 | 30.5 | 1.11 | 1.32 | 0.32 | 6 | 26.47 | 1.96 | 1.335 | 0.48 |
| NSW | Temperate | 662 | P | Gripopterygidae | Dinotoperla | Dinotoperla christinae | 10 | 1.17 | 1.15 | 0.96 | 0.1 | 9 | 29.33 | 1.95 | 0.94 | 0.09 | 19 | 28.16 | 3.1 | 0.95 | 0.19 |
| NSW | Temperate | 662 | P | Gripopterygidae | Trinotoperla | Trinotoperla sp | 4 | 3.85 | 0.26 | 1.25 | 0.31 | 6 | 27.52 | 2.36 | 1.06 | 0.22 | 10 | 23.67 | 2.62 | 1.155 | 0.53 |
| NSW | Temperate | 662 | T | Hydrobiosidae | Ulmerochorema | Ulmerochorema rubiconum | 3 | 3.33 | 0.32 | 1.16 | 0.08 | NA | NA | NA | NA | NA | NA | NA | NA | NA | NA |
| NSW | Temperate | 662 | T | Hydropsychidae | Asmicridea | Asmicridea sp AV1 | 9 | 4.54 | 0.79 | 1.63 | 0.08 | 10 | 30.4 | 3.14 | 1.55 | 0.09 | 19 | 25.86 | 3.93 | 1.59 | 0.17 |
| NSW | Temperate | 662 | T | Hydropsychidae | Diplectrona | Diplectrona sp | NA | NA | NA | NA | NA | 3 | 28.43 | 3.35 | 1.94 | 0.22 | NA | NA | NA | NA | NA |
| NSW | Temperate | 662 | T | Philopotamidae | Chimarra | Chimarra monticola | 7 | 4.8 | 0.63 | 1.19 | 0.03 | 9 | 29.77 | 0.69 | 1.18 | 0.03 | 16 | 24.97 | 1.32 | 1.185 | 0.06 |
| NSW | Temperate | 662 | T | Philorheithridae | Kosrheithrus | Kosrheithrus tillyardi | NA | NA | NA | NA | NA | 3 | 29.53 | 2.37 | 1.55 | 0.39 | NA | NA | NA | NA | NA |
| NSW | Temperate | 796 | E | Baetidae | Offadens | Offadens o hickmani complex | 9 | 2.9 | 0.87 | 1.41 | 0.12 | 10 | 27.66 | 1.24 | 1.33 | 0.12 | 19 | 24.76 | 2.11 | 1.37 | 0.24 |
| NSW | Temperate | 796 | E | Coloburiscidae | Coloburiscoides | Coloburiscoides giganteus | 7 | 2.44 | 0.54 | 1.77 | 0.69 | 6 | 31.15 | 1.58 | 1.78 | 0.5 | 13 | 28.71 | 2.12 | 1.775 | 1.19 |
| NSW | Temperate | 796 | E | Leptophlebiidae | Atalophlebia | Atalophlebia sp AV4 | NA | NA | NA | NA | NA | 6 | 31.75 | 1.8 | 2.01 | 0.39 | NA | NA | NA | NA | NA |
| NSW | Temperate | 796 | E | Leptophlebiidae | Austrophlebioides | Austrophlebioides marchanti sp | 6 | 1.95 | 0.47 | 2.48 | 0.15 | 9 | 31.62 | 1.52 | 2.38 | 0.07 | 15 | 29.67 | 1.99 | 2.43 | 0.22 |
| NSW | Temperate | 796 | E | Leptophlebiidae | Nousia | Nousia sp AV11 | 4 | 1.48 | 0.87 | 2.24 | 0.11 | NA | NA | NA | NA | NA | NA | NA | NA | NA | NA |
| NSW | Temperate | 796 | E | Leptophlebiidae | Nousia | Nousia sp AV2 | 3 | 0.6 | 0.26 | 2.31 | 0.19 | 3 | 30.13 | 2.42 | 2.35 | 0.12 | 6 | 29.53 | 2.68 | 2.33 | 0.31 |
| NSW | Temperate | 796 | P | Austroperlidae | Austroheptura | Austroheptura sp 2 | 4 | 0.92 | 1.26 | 1.94 | 0.51 | 4 | 32.77 | 1.02 | 2.03 | 0.16 | 8 | 31.85 | 2.28 | 1.985 | 0.67 |
| NSW | Temperate | 796 | P | Eustheniidae | Cosmioperla | Cosmioperla kuna | 5 | 0.64 | 1.16 | 3.59 | 1.7 | 6 | 33.65 | 0.71 | 3.31 | 1.42 | 11 | 33.01 | 1.87 | 3.45 | 3.12 |
| NSW | Temperate | 796 | P | Gripopterygidae | Dinotoperla | Dinotoperla eucumbene | 5 | 2.9 | 1.51 | 0.94 | 0.06 | 6 | 30.85 | 1.54 | 0.87 | 0.06 | 11 | 27.95 | 3.05 | 0.905 | 0.12 |
| NSW | Temperate | 796 | P | Gripopterygidae | Dinotoperla | Dinotoperla serricauda thwaitesi | NA | NA | NA | NA | NA | 3 | 33.57 | 0.5 | 0.89 | 0.05 | NA | NA | NA | NA | NA |
| NSW | Temperate | 796 | P | Gripopterygidae | Trinotoperla | Trinotoperla sp | 5 | 4.54 | 0.4 | 0.82 | 0.18 | 7 | 29.87 | 2.43 | 0.81 | 0.15 | 12 | 25.33 | 2.83 | 0.815 | 0.33 |
| NSW | Temperate | 796 | T | Calamoceratidae | Anisocentropus | Anisocentropus sp | NA | NA | NA | NA | NA | 7 | 28.53 | 1.05 | 0.74 | 0.21 | NA | NA | NA | NA | NA |
| NSW | Temperate | 796 | T | Calocidae | Tamasia | Tamasia acuta | 9 | 4.73 | 0.68 | 0.98 | 0.2 | 9 | 28.16 | 2.38 | 1 | 0.15 | 18 | 23.43 | 3.06 | 0.99 | 0.35 |
| NSW | Temperate | 796 | T | Hydrobiosidae | Taschorema complex | Taschorema evansi | 3 | 0.37 | 0.15 | 1.9 | 0.03 | 4 | 28.02 | 1.05 | 1.78 | 0.13 | 7 | 27.65 | 1.2 | 1.84 | 0.16 |
| NSW | Temperate | 796 | T | Hydropsychidae | Asmicridea | Asmicridea sp AV1 | 7 | 1.61 | 0.56 | 1.68 | 0.04 | 9 | 32.16 | 1.57 | 1.5 | 0.26 | 16 | 30.55 | 2.13 | 1.59 | 0.3 |
| NSW | Temperate | 796 | T | Hydropsychidae | Cheumatopsyche | Cheumatopsyche sp AV3 | 3 | 1.63 | 0.32 | 1.24 | 0.08 | NA | NA | NA | NA | NA | NA | NA | NA | NA | NA |
| NSW | Temperate | 796 | T | Leptoceridae | Triplectides | Triplectides proximus | 4 | 4.88 | 0.33 | 0.65 | 0.05 | NA | NA | NA | NA | NA | NA | NA | NA | NA | NA |
| NSW | Temperate | 796 | T | Leptoceridae | Triplectides | Triplectides proximus sp | 4 | 5.45 | 0.56 | 1.06 | 0.28 | 7 | 30.69 | 1.13 | 1.12 | 0.27 | 11 | 25.24 | 1.69 | 1.09 | 0.55 |
| NSW | Temperate | 796 | T | Philopotamidae | Chimarra | Chimarra monticola | 3 | 3.17 | 1.82 | 1.21 | 0.06 | 4 | 31.15 | 0.92 | 1.14 | 0.06 | 7 | 27.98 | 2.74 | 1.175 | 0.12 |
| NSW | Temperate | 796 | T | Philorheithridae | Aphilorheithrus | Aphilorheithrus spp | NA | NA | NA | NA | NA | 3 | 28.03 | 1.36 | 1.1 | 0.2 | NA | NA | NA | NA | NA |
| NSW | Temperate | 1140 | E | Baetidae | Offadens | Offadens o hickmani complex | 14 | 2.71 | 0.55 | 1.15 | 0.1 | 9 | 29.52 | 0.33 | 1.16 | 0.09 | 23 | 26.81 | 0.88 | 1.155 | 0.19 |
| NSW | Temperate | 1140 | E | Leptophlebiidae | Atalophlebia | Atalophlebia sp AV4 | 3 | 2 | 0.3 | 2.21 | 0.57 | 3 | 34.5 | 0.3 | 1.78 | 0.25 | 6 | 32.5 | 0.6 | 1.995 | 0.82 |
| NSW | Temperate | 1140 | E | Leptophlebiidae | Austrophlebioides | Austrophlebioides marchanti | NA | NA | NA | NA | NA | 3 | 32.87 | 3.87 | 2.2 | 0.11 | NA | NA | NA | NA | NA |
| NSW | Temperate | 1140 | E | Leptophlebiidae | Austrophlebioides | Austrophlebioides marchanti sp | 3 | 2.7 | 0.17 | 2.57 | 0.19 | 3 | 32.13 | 0.06 | 2.5 | 0.08 | 6 | 29.43 | 0.23 | 2.535 | 0.27 |
| NSW | Temperate | 1140 | E | Leptophlebiidae | Austrophlebioides | Austrophlebioides pusillus | 4 | 2.2 | 0.18 | 1.79 | 0.27 | NA | NA | NA | NA | NA | NA | NA | NA | NA | NA |
| NSW | Temperate | 1140 | E | Leptophlebiidae | Nousia | Nousia sp AV1 | 5 | 2.36 | 0.15 | 1.57 | 0.17 | 8 | 33.27 | 0.95 | 1.62 | 0.08 | 13 | 30.91 | 1.1 | 1.595 | 0.25 |
| NSW | Temperate | 1140 | P | Gripopterygidae | Dinotoperla | Dinotoperla christinae | 9 | 1.06 | 0.82 | 1.06 | 0.13 | 10 | 32.13 | 1.47 | 1.02 | 0.12 | 19 | 31.07 | 2.29 | 1.04 | 0.25 |
| NSW | Temperate | 1140 | P | Gripopterygidae | Riekoperla | Riekoperla rugosa group | 4 | 1.22 | 0.79 | 1.21 | 0.04 | 5 | 33.28 | 1.62 | 1.14 | 0.11 | 9 | 32.06 | 2.41 | 1.175 | 0.15 |
| NSW | Temperate | 1140 | T | Calamoceratidae | Anisocentropus | Anisocentropus sp | NA | NA | NA | NA | NA | 8 | 30.76 | 1.7 | 0.91 | 0.15 | NA | NA | NA | NA | NA |
| NSW | Temperate | 1140 | T | Calocidae | Tamasia | Tamasia acuta | 7 | 3.5 | 1.23 | 1.07 | 0.13 | 4 | 29.02 | 1.58 | 0.89 | 0.23 | 11 | 25.52 | 2.81 | 0.98 | 0.36 |
| NSW | Temperate | 1140 | T | Conoesucidae | Coenoria | Coenoria sp AV4 | 3 | 3.17 | 0.47 | 1.01 | 0.13 | NA | NA | NA | NA | NA | NA | NA | NA | NA | NA |
| NSW | Temperate | 1140 | T | Conoesucidae | Conoesucus | Conoesucus sp AV2 | 5 | 4.58 | 2.27 | 0.82 | 0.1 | 8 | 31.2 | 2.63 | 0.78 | 0.15 | 13 | 26.62 | 4.9 | 0.8 | 0.25 |
| NSW | Temperate | 1140 | T | Hydrobiosidae | Apsilochorema | Apsilochorema obliquum | NA | NA | NA | NA | NA | 3 | 27.73 | 1.78 | 1.14 | 0.04 | NA | NA | NA | NA | NA |
| NSW | Temperate | 1140 | T | Hydrobiosidae | Taschorema complex | Taschorema evansi | 3 | 2.57 | 2.12 | 1.11 | 0.33 | 3 | 29.87 | 0.55 | 1.12 | 0.14 | 6 | 27.3 | 2.67 | 1.115 | 0.47 |
| NSW | Temperate | 1140 | T | Hydropsychidae | Asmicridea | Asmicridea sp AV1 | 8 | 2.91 | 1.46 | 1.61 | 0.19 | 8 | 33.19 | 2.57 | 1.58 | 0.09 | 16 | 30.28 | 4.03 | 1.595 | 0.28 |
| NSW | Temperate | 1140 | T | Leptoceridae | Triplectides | Triplectides ciuskus ciuskus | NA | NA | NA | NA | NA | 3 | 34 | 3.17 | 1.08 | 0.49 | NA | NA | NA | NA | NA |
| NSW | Temperate | 1140 | T | Leptoceridae | Triplectides | Triplectides proximus | NA | NA | NA | NA | NA | 4 | 30.1 | 0.64 | 0.81 | 0.17 | NA | NA | NA | NA | NA |
| NSW | Temperate | 1140 | T | Philopotamidae | Chimarra | Chimarra monticola | 9 | 2.81 | 0.29 | 1.19 | 0.06 | 9 | 30.79 | 0.93 | 1.18 | 0.06 | 18 | 27.98 | 1.22 | 1.185 | 0.12 |
| NSW | Temperate | 1140 | T | Philorheithridae | Aphilorheithrus | Aphilorheithrus sp | NA | NA | NA | NA | NA | 3 | 28.73 | 1.07 | 1.33 | 0.31 | NA | NA | NA | NA | NA |
| NSW | Temperate | 1140 | T | Philorheithridae | Aphilorheithrus | Aphilorheithrus spp AV3 | NA | NA | NA | NA | NA | 3 | 30.47 | 2.46 | 0.62 | 0.22 | NA | NA | NA | NA | NA |
| NSW | Temperate | 1140 | T | Philorheithridae | Kosrheithrus | Kosrheithrus tillyardi | 5 | 4.12 | 1.48 | 1.42 | 0.87 | 3 | 30 | 2.59 | 1.03 | 0.84 | 8 | 25.88 | 4.07 | 1.225 | 1.71 |
| NSW | Temperate | 1341 | E | Baetidae | Offadens | Offadens o hickmani complex | 9 | 3.29 | 0.36 | 1.45 | 0.1 | 9 | 27.06 | 1.67 | 1.4 | 0.06 | 18 | 23.77 | 2.03 | 1.425 | 0.16 |
| NSW | Temperate | 1341 | E | Coloburiscidae | Coloburiscoides | Coloburiscoides giganteus | NA | NA | NA | NA | NA | 3 | 30.6 | 1.57 | 2.1 | 0.62 | NA | NA | NA | NA | NA |
| NSW | Temperate | 1341 | E | Leptophlebiidae | Austrophlebioides | Austrophlebioides marchanti sp | 10 | 2.83 | 0.56 | 2.63 | 0.24 | 9 | 31.52 | 0.89 | 2.65 | 0.16 | 19 | 28.69 | 1.45 | 2.64 | 0.4 |
| NSW | Temperate | 1341 | P | Eustheniidae | Cosmioperla | Cosmioperla kuna | NA | NA | NA | NA | NA | 5 | 31.04 | 2.58 | 2.77 | 1.48 | NA | NA | NA | NA | NA |
| NSW | Temperate | 1341 | P | Gripopterygidae | Dinotoperla | Dinotoperla eucumbene | 9 | 4.59 | 0.5 | 0.89 | 0.06 | 8 | 33.49 | 0.86 | 0.93 | 0.07 | 17 | 28.9 | 1.36 | 0.91 | 0.13 |
| NSW | Temperate | 1341 | P | Gripopterygidae | Trinotoperla | Trinotoperla sp | 9 | 0.7 | 0.53 | 1.56 | 0.24 | 9 | 33.06 | 0.66 | 1.44 | 0.22 | 18 | 32.36 | 1.19 | 1.5 | 0.46 |
| NSW | Temperate | 1341 | T | Calocidae | Tamasia | Tamasia acuta | 6 | 4.5 | 0.72 | 0.89 | 0.26 | 5 | 29.38 | 0.9 | 1.14 | 0.12 | 11 | 24.88 | 1.62 | 1.015 | 0.38 |
| NSW | Temperate | 1341 | T | Conoesucidae | Conoesucus | Conoesucus sp AV2 | 8 | 5.19 | 0.7 | 1.28 | 0.12 | 10 | 32.38 | 3.86 | 1.23 | 0.19 | 18 | 27.19 | 4.56 | 1.255 | 0.31 |
| NSW | Temperate | 1341 | T | Hydrobiosidae | Taschorema complex | Taschorema evansi | 9 | 2.76 | 1.19 | 1.66 | 0.33 | 8 | 27.73 | 0.48 | 1.54 | 0.35 | 17 | 24.97 | 1.67 | 1.6 | 0.68 |
| NSW | Temperate | 1341 | T | Hydropsychidae | Asmicridea | Asmicridea sp AV1 | NA | NA | NA | NA | NA | 3 | 32.63 | 0.91 | 1.44 | 0.58 | NA | NA | NA | NA | NA |
| NSW | Temperate | 1341 | T | Philorheithridae | Kosrheithrus | Kosrheithrus tillyardi | 9 | 2.77 | 1.33 | 1.64 | 0.73 | 8 | 31.34 | 1.62 | 1.95 | 0.49 | 17 | 28.57 | 2.95 | 1.795 | 1.22 |
| NSW | Temperate | 1500 | E | Baetidae | Offadens | Offadens o hickmani complex | 18 | 3.41 | 0.45 | 1.09 | 0.06 | 19 | 29.54 | 0.7 | 1.08 | 0.06 | 37 | 26.13 | 1.15 | 1.085 | 0.12 |
| NSW | Temperate | 1500 | E | Coloburiscidae | Coloburiscoides | Coloburiscoides giganteus | 9 | 1.96 | 0.44 | 3.09 | 0.9 | 9 | 33.56 | 0.78 | 2.97 | 0.74 | 18 | 31.6 | 1.22 | 3.03 | 1.64 |
| NSW | Temperate | 1500 | E | Leptophlebiidae | Austrophlebioides | Austrophlebioides marchanti | 7 | 3.64 | 0.26 | 1.93 | 0.54 | 6 | 31.95 | 1.16 | 1.7 | 0.29 | 13 | 28.31 | 1.42 | 1.815 | 0.83 |
| NSW | Temperate | 1500 | E | Leptophlebiidae | Nousia | Nousia spp AV11 | 9 | 1.99 | 0.56 | 2.47 | 0.15 | 13 | 31.92 | 0.85 | 2.44 | 0.19 | 22 | 29.93 | 1.41 | 2.455 | 0.34 |
| NSW | Temperate | 1500 | P | Austroperlidae | Austroheptura | Austroheptura sp 2 | 6 | 1.1 | 0.65 | 1.58 | 0.61 | 5 | 33.46 | 1.28 | 1.51 | 0.43 | 11 | 32.36 | 1.93 | 1.545 | 1.04 |
| NSW | Temperate | 1500 | P | Eustheniidae | Cosmioperla | Cosmioperla kuna | 9 | 0.1 | 0.21 | 3.87 | 0.93 | 9 | 33.9 | 1.77 | 3.64 | 0.92 | 18 | 33.8 | 1.98 | 3.755 | 1.85 |
| NSW | Temperate | 1500 | P | Gripopterygidae | Eunotoperla | Eunotoperla sp | NA | NA | NA | NA | NA | 4 | 32.83 | 0.51 | 1.37 | 0.19 | NA | NA | NA | NA | NA |
| NSW | Temperate | 1500 | P | Gripopterygidae | Trinotoperla | Trinotoperla sp | 4 | 0.32 | 0.1 | 1.3 | 0.17 | NA | NA | NA | NA | NA | NA | NA | NA | NA | NA |
| NSW | Temperate | 1500 | P | Notonemouridae | Austrocercella | Austrocercella spp | NA | NA | NA | NA | NA | 4 | 30.75 | 0.21 | 1.05 | 0.14 | NA | NA | NA | NA | NA |
| NSW | Temperate | 1500 | T | Calocidae | Tamasia | Tamasia acuta | 9 | 3.7 | 0.5 | 0.98 | 0.06 | 8 | 32.17 | 0.55 | 0.97 | 0.09 | 17 | 28.47 | 1.05 | 0.975 | 0.15 |
| NSW | Temperate | 1500 | T | Conoesucidae | Conoesucus | Conoesucus sp AV2 | 9 | 2.58 | 1.19 | 0.77 | 0.1 | 9 | 33.28 | 1.42 | 0.82 | 0.12 | 18 | 30.7 | 2.61 | 0.795 | 0.22 |
| NSW | Temperate | 1500 | T | Hydrobiosidae | Taschorema complex | Taschorema evansi | 5 | 2.42 | 0.26 | 1.47 | 0.4 | 5 | 30.22 | 0.51 | 1.21 | 0.49 | 10 | 27.8 | 0.77 | 1.34 | 0.89 |
| NSW | Temperate | 1500 | T | Hydropsychidae | Asmicridea | Asmicridea sp AV1 | 7 | 3 | 0.95 | 1.72 | 0.09 | 8 | 33.26 | 0.47 | 1.63 | 0.11 | 15 | 30.26 | 1.42 | 1.675 | 0.2 |
| NSW | Temperate | 1500 | T | Philopotamidae | Chimarra | Chimarra monticola | NA | NA | NA | NA | NA | 3 | 29.6 | 0.36 | 1.08 | 0.08 | NA | NA | NA | NA | NA |
| NSW | Temperate | 1500 | T | Philorheithridae | Austrheithrus | Austrheithrus sp | 3 | 2.4 | 0.75 | 1.23 | 0.13 | NA | NA | NA | NA | NA | NA | NA | NA | NA | NA |
| NSW | Temperate | 1500 | T | Philorheithridae | Kosrheithrus | Kosrheithrus tillyardi | NA | NA | NA | NA | NA | 3 | 33.73 | 1.27 | 1.85 | 0.42 | NA | NA | NA | NA | NA |
| NSW | Temperate | 1710 | E | Baetidae | Offadens | Offadens o hickmani complex | 8 | 3.2 | 1.04 | 1.21 | 0.1 | 7 | 27.57 | 1.05 | 1.27 | 0.07 | 15 | 24.37 | 2.09 | 1.24 | 0.17 |
| NSW | Temperate | 1710 | E | Coloburiscidae | Coloburiscoides | Coloburiscoides giganteus | 9 | 1.51 | 0.56 | 2.8 | 0.54 | 9 | 30.22 | 2.03 | 3.09 | 0.57 | 18 | 28.71 | 2.59 | 2.945 | 1.11 |
| NSW | Temperate | 1710 | E | Leptophlebiidae | Austrophlebioides | Austrophlebioides sp AV2 | 5 | 3.24 | 0.72 | 2.3 | 0.23 | 15 | 32.97 | 1.22 | 2.22 | 0.26 | 20 | 29.73 | 1.94 | 2.26 | 0.49 |
| NSW | Temperate | 1710 | E | Leptophlebiidae | Nousia | Nousia sp AV11 | 13 | 1.47 | 0.43 | 2.19 | 0.25 | 15 | 31.46 | 0.97 | 2.06 | 0.21 | 28 | 29.99 | 1.4 | 2.125 | 0.46 |
| NSW | Temperate | 1710 | P | Austroperlidae | Austroheptura | Austroheptura sp 2 | 9 | 1.66 | 1.3 | 1.65 | 0.27 | 9 | 32.73 | 0.81 | 1.49 | 0.4 | 18 | 31.07 | 2.11 | 1.57 | 0.67 |
| NSW | Temperate | 1710 | P | Eustheniidae | Cosmioperla | Cosmioperla kuna | 7 | 0.5 | 0.61 | 3.72 | 1.15 | 8 | 34.15 | 0.8 | 4.27 | 0.84 | 15 | 33.65 | 1.41 | 3.995 | 1.99 |
| NSW | Temperate | 1710 | P | Gripopterygidae | Dinotoperla | Dinotoperla eucumbene | 9 | 1.48 | 0.68 | 0.92 | 0.07 | 9 | 33.22 | 1.84 | 0.88 | 0.09 | 18 | 31.74 | 2.52 | 0.9 | 0.16 |
| NSW | Temperate | 1710 | P | Gripopterygidae | Riekoperla | Riekoperla alpina | 4 | 0.92 | 0.92 | 0.93 | 0.14 | 3 | 33.27 | 0.49 | 0.92 | 0.02 | 7 | 32.35 | 1.41 | 0.925 | 0.16 |
| NSW | Temperate | 1710 | P | Gripopterygidae | Riekoperla | Riekoperla rugosa group | 3 | 0.83 | 0.7 | 0.97 | 0.22 | NA | NA | NA | NA | NA | NA | NA | NA | NA | NA |
| NSW | Temperate | 1710 | T | Conoesucidae | Conoesucus | Conoesucus sp AV2 | 8 | 3.28 | 0.55 | 1.08 | 0.19 | 9 | 33.58 | 1.3 | 1.03 | 0.14 | 17 | 30.31 | 1.85 | 1.055 | 0.33 |
| NSW | Temperate | 1710 | T | Hydrobiosidae | Taschorema complex | Taschorema evansi | 7 | 1.91 | 1.66 | 1.36 | 0.47 | 8 | 29.82 | 0.65 | 1.14 | 0.38 | 15 | 27.91 | 2.31 | 1.25 | 0.85 |
| NSW | Temperate | 1710 | T | Hydropsychidae | Asmicridea | Asmicridea sp AV1 | NA | NA | NA | NA | NA | 3 | 34.2 | 0.62 | 1.82 | 0.04 | NA | NA | NA | NA | NA |
| NSW | Temperate | 1710 | T | Philorheithridae | Kosrheithrus | Kosrheithrus tillyardi | 6 | 1.42 | 0.42 | 1.91 | 0.26 | 7 | 31.97 | 1.43 | 1.97 | 0.34 | 13 | 30.55 | 1.85 | 1.94 | 0.6 |
| NSW | Temperate | 1943 | E | Baetidae | Offadens | Offadens o hickmani complex | 13 | 3.47 | 0.78 | 1.17 | 0.14 | 12 | 31.27 | 1.39 | 1.14 | 0.2 | 25 | 27.8 | 2.17 | 1.155 | 0.34 |
| NSW | Temperate | 1943 | E | Coloburiscidae | Coloburiscoides | Coloburiscoides giganteus | 9 | 1.98 | 0.56 | 2.65 | 0.56 | 9 | 35.19 | 0.78 | 2.75 | 0.45 | 18 | 33.21 | 1.34 | 2.7 | 1.01 |
| NSW | Temperate | 1943 | E | Leptophlebiidae | Atalophlebia | Atalophlebia albiterminata | 13 | 2.42 | 0.51 | 1.45 | 0.2 | 14 | 34.99 | 2.76 | 1.28 | 0.16 | 27 | 32.57 | 3.27 | 1.365 | 0.36 |
| NSW | Temperate | 1943 | E | Leptophlebiidae | Atalophlebia | Atalophlebia sp AV21 | NA | NA | NA | NA | NA | 3 | 32.4 | 1.61 | 0.99 | 0.11 | NA | NA | NA | NA | NA |
| NSW | Temperate | 1943 | E | Leptophlebiidae | Austrophlebioides | Austrophlebioides marchanti | 4 | 2.25 | 0.34 | 1.76 | 0.42 | NA | NA | NA | NA | NA | NA | NA | NA | NA | NA |
| NSW | Temperate | 1943 | E | Leptophlebiidae | Austrophlebioides | Austrophlebioides pusillus | 3 | 2.83 | 0.8 | 2.43 | 0.98 | 5 | 35.82 | 0.9 | 1.76 | 0.13 | 8 | 32.99 | 1.7 | 2.095 | 1.11 |
| NSW | Temperate | 1943 | E | Leptophlebiidae | Austrophlebioides | Austrophlebioides sp AV2 | 3 | 3.13 | 0.12 | 1.99 | 0.17 | 4 | 34.98 | 0.78 | 1.84 | 0.11 | 7 | 31.85 | 0.9 | 1.915 | 0.28 |
| NSW | Temperate | 1943 | P | Eustheniidae | Cosmioperla | Cosmioperla kuna | 9 | 0.17 | 0.46 | 4.68 | 2.01 | 9 | 35.73 | 0.41 | 3.78 | 0.86 | 18 | 35.56 | 0.87 | 4.23 | 2.87 |
| NSW | Temperate | 1943 | P | Gripopterygidae | Dinotoperla | Dinotoperla fontana | 9 | 2.96 | 0.58 | 0.73 | 0.11 | 12 | 33.76 | 1.42 | 0.87 | 0.23 | 21 | 30.8 | 2 | 0.8 | 0.34 |
| NSW | Temperate | 1943 | T | Conoesucidae | Coenoria | Coenoria sp AV4 | 9 | 4.69 | 0.67 | 0.73 | 0.08 | 9 | 33.33 | 1.89 | 0.71 | 0.05 | 18 | 28.64 | 2.56 | 0.72 | 0.13 |
| NSW | Temperate | 1943 | T | Hydrobiosidae | Taschorema complex | Taschorema evansi | 7 | 2.11 | 0.63 | 1.29 | 0.39 | 6 | 28.85 | 0.97 | 1.3 | 0.49 | 13 | 26.74 | 1.6 | 1.295 | 0.88 |
| NSW | Temperate | 1943 | T | Hydropsychidae | Asmicridea | Asmicridea sp AV1 | 9 | 1.53 | 0.49 | 1.57 | 0.23 | 9 | 30.41 | 0.69 | 1.69 | 0.2 | 18 | 28.88 | 1.18 | 1.63 | 0.43 |
| NSW | Temperate | 1943 | T | Leptoceridae | Notalina | Notalina fulva | 8 | 3.35 | 0.42 | 0.5 | 0.04 | 3 | 32.73 | 2.76 | 0.35 | 0.03 | 11 | 29.38 | 3.18 | 0.425 | 0.07 |
| NSW | Temperate | 1943 | T | Philopotamidae | Chimarra | Chimarra monticola | 9 | 4.1 | 0.18 | 1.14 | 0.08 | 9 | 32.13 | 2.2 | 1.28 | 0.28 | 18 | 28.03 | 2.38 | 1.21 | 0.36 |
| NSW | Temperate | 1943 | T | Philorheithridae | Kosrheithrus | Kosrheithrus tillyardi | 9 | 0.87 | 0.68 | 1.08 | 0.37 | 8 | 31.17 | 2.97 | 1.3 | 0.61 | 17 | 30.3 | 3.65 | 1.19 | 0.98 |
| QLD | Tropical | 30 | E | Baetidae | Offadens | Offadens o hickmani complex qld | 11 | 7.72 | 1.21 | 0.79 | 0.1 | 11 | 33.12 | 0.73 | 0.82 | 0.13 | 22 | 25.4 | 1.94 | 0.805 | 0.23 |
| QLD | Tropical | 30 | E | Leptophlebiidae | Atalomicria | Atalomicria sexfasciata sp A | 6 | 7.65 | 0.93 | 1.33 | 0.15 | 9 | 33.51 | 1.07 | 1.18 | 0.24 | 15 | 25.86 | 2 | 1.255 | 0.39 |
| QLD | Tropical | 30 | E | Leptophlebiidae | Austrophlebioides | Austrophlebioides rieki | 7 | 6.91 | 1.08 | 1.69 | 0.21 | 6 | 34.22 | 1.36 | 1.55 | 0.24 | 13 | 27.31 | 2.44 | 1.62 | 0.45 |
| QLD | Tropical | 30 | E | Leptophlebiidae | Austrophlebioides | Austrophlebioides rieki sp | 6 | 6.58 | 1.43 | 1.75 | 0.06 | 6 | 34.77 | 1.6 | 1.63 | 0.1 | 12 | 28.19 | 3.03 | 1.69 | 0.16 |
| QLD | Tropical | 30 | E | Leptophlebiidae | Austrophlebioides | Austrophlebioides rieki spp | 9 | 7.57 | 0.54 | 1.72 | 0.16 | 9 | 34.73 | 1.22 | 1.59 | 0.08 | 18 | 27.16 | 1.76 | 1.655 | 0.24 |
| QLD | Tropical | 30 | E | Leptophlebiidae | Nousia | Nousia sp A | NA | NA | NA | NA | NA | 3 | 33.63 | 0.81 | 1.35 | 0.12 | NA | NA | NA | NA | NA |
| QLD | Tropical | 30 | E | Leptophlebiidae | Nousia | Nousia sp B | 3 | 7.4 | 0.26 | 1.1 | 0.24 | 3 | 33.37 | 1.58 | 1.25 | 0.16 | 6 | 25.97 | 1.84 | 1.175 | 0.4 |
| QLD | Tropical | 30 | P | Eustheniidae | Cosmioperla | Cosmioperla kuna qld | 3 | 4.1 | 0.46 | 2.72 | 0.45 | 4 | 35.23 | 0.53 | 1.52 | 0.68 | 7 | 31.13 | 0.99 | 2.12 | 1.13 |
| QLD | Tropical | 30 | P | Gripopterygidae | Illisioperla | Illisioperla barbara | 5 | 7.84 | 2.66 | 0.82 | 0.14 | 9 | 34.64 | 0.41 | 1.01 | 0.22 | 14 | 26.8 | 3.07 | 0.915 | 0.36 |
| QLD | Tropical | 30 | P | Gripopterygidae | Illisioperla | Illisioperla cerberus | 4 | 8.53 | 3.85 | 1.15 | 0.2 | NA | NA | NA | NA | NA | NA | NA | NA | NA | NA |
| QLD | Tropical | 30 | P | Gripopterygidae | Trinotoperla | Trinotoperla sp A | 9 | 8.62 | 1.58 | 0.95 | 0.2 | 10 | 33.73 | 1.86 | 1.03 | 0.15 | 19 | 25.11 | 3.44 | 0.99 | 0.35 |
| QLD | Tropical | 30 | T | Calocidae | Calocoides | Calocoides aquilona | 3 | 4.87 | 0.29 | 0.71 | 0.06 | NA | NA | NA | NA | NA | NA | NA | NA | NA | NA |
| QLD | Tropical | 30 | T | Hydrobiosidae | Taschorema complex | Taschorema evansi qld | 3 | 6.9 | 1.06 | 0.86 | 0.25 | 3 | 32.03 | 0.4 | 0.95 | 0.26 | 6 | 25.13 | 1.46 | 0.905 | 0.51 |
| QLD | Tropical | 30 | T | Hydropsychidae | Asmicridea | Asmicridea sp D | 13 | 7.31 | 1.54 | 0.89 | 0.14 | 15 | 34.51 | 1.82 | 0.96 | 0.17 | 28 | 27.2 | 3.36 | 0.925 | 0.31 |
| QLD | Tropical | 30 | T | Hydropsychidae | Asmicridea | Asmicridea sp E | 6 | 6.58 | 0.88 | 1.04 | 0.12 | 3 | 34.97 | 1.1 | 0.89 | 0.13 | 9 | 28.39 | 1.98 | 0.965 | 0.25 |
| QLD | Tropical | 30 | T | Philopotamidae | Chimarra | Chimarra sp au | 5 | 8.48 | 0.33 | 0.67 | 0.03 | 7 | 32.44 | 1.5 | 0.68 | 0.03 | 12 | 23.96 | 1.83 | 0.675 | 0.06 |
| QLD | Tropical | 30 | T | Philopotamidae | Chimarra | Chimarra sp du2t | 3 | 8.87 | 0.31 | 0.81 | 0.04 | 3 | 33.37 | 0.81 | 0.81 | 0.04 | 6 | 24.5 | 1.12 | 0.81 | 0.08 |
| QLD | Tropical | 97 | E | Baetidae | Offadens | Offadens o hickmani complex qld | 4 | 8.5 | 1.41 | 0.8 | 0.13 | 4 | 34.15 | 1.54 | 0.82 | 0.12 | 8 | 25.65 | 2.95 | 0.81 | 0.25 |
| QLD | Tropical | 97 | E | Leptophlebiidae | Austrophlebioides | Austrophlebioides rieki | 8 | 7.34 | 0.46 | 1.77 | 0.39 | 7 | 35.53 | 0.56 | 1.64 | 0.32 | 15 | 28.19 | 1.02 | 1.705 | 0.71 |
| QLD | Tropical | 97 | E | Leptophlebiidae | Austrophlebioides | Austrophlebioides rieki sp | 5 | 7.22 | 0.25 | 1.89 | 0.25 | 10 | 35.49 | 0.55 | 1.62 | 0.17 | 15 | 28.27 | 0.8 | 1.755 | 0.42 |
| QLD | Tropical | 97 | E | Leptophlebiidae | Austrophlebioides | Austrophlebioides rieki spp | 7 | 7.13 | 0.93 | 1.85 | 0.31 | 4 | 35.27 | 0.95 | 1.35 | 0.14 | 11 | 28.14 | 1.88 | 1.6 | 0.45 |
| QLD | Tropical | 97 | E | Leptophlebiidae | Nousia | Nousia sp A | NA | NA | NA | NA | NA | 3 | 34.87 | 0.47 | 1.16 | 0.14 | NA | NA | NA | NA | NA |
| QLD | Tropical | 97 | E | Leptophlebiidae | Nousia | Nousia sp B | 6 | 6.45 | 0.85 | 1.12 | 0.06 | 7 | 34.99 | 0.51 | 1.15 | 0.16 | 13 | 28.54 | 1.36 | 1.135 | 0.22 |
| QLD | Tropical | 97 | E | Leptophlebiidae | Nousia | Nousia sp C | 4 | 5.85 | 0.37 | 1.43 | 0.2 | 4 | 34.73 | 0.71 | 1.35 | 0.14 | 8 | 28.88 | 1.08 | 1.39 | 0.34 |
| QLD | Tropical | 97 | P | Gripopterygidae | Illisioperla | Illisioperla barbara | 12 | 7.69 | 1.6 | 0.88 | 0.28 | 10 | 34.24 | 1.12 | 0.89 | 0.23 | 22 | 26.55 | 2.72 | 0.885 | 0.51 |
| QLD | Tropical | 97 | P | Gripopterygidae | Illisioperla | Illisioperla cerberus | NA | NA | NA | NA | NA | 3 | 34.53 | 0.76 | 1.24 | 0.17 | NA | NA | NA | NA | NA |
| QLD | Tropical | 97 | T | Calamoceratidae | Anisocentropus | Anisocentropus sp qld | NA | NA | NA | NA | NA | 10 | 34.94 | 1.51 | 0.65 | 0.2 | NA | NA | NA | NA | NA |
| QLD | Tropical | 97 | T | Hydropsychidae | Asmicridea | Asmicridea sp C | 5 | 7.46 | 1.23 | 0.95 | 0.15 | 4 | 34.98 | 1.44 | 1.01 | 0.13 | 9 | 27.52 | 2.67 | 0.98 | 0.28 |
| QLD | Tropical | 97 | T | Hydropsychidae | Asmicridea | Asmicridea sp D | 11 | 8.17 | 2.01 | 0.96 | 0.15 | 14 | 35.05 | 1.43 | 0.83 | 0.17 | 25 | 26.88 | 3.44 | 0.895 | 0.32 |
| QLD | Tropical | 97 | T | Hydropsychidae | Asmicridea | Asmicridea sp E | 5 | 10.06 | 1.64 | 0.79 | 0.01 | 3 | 35.83 | 0.8 | 0.89 | 0.14 | 8 | 25.77 | 2.44 | 0.84 | 0.15 |
| QLD | Tropical | 97 | T | Odontoceridae | Barynema | Barynema sp AV1 | NA | NA | NA | NA | NA | 4 | 34.35 | 0.75 | 1.31 | 0.24 | NA | NA | NA | NA | NA |
| QLD | Tropical | 97 | T | Philopotamidae | Chimarra | Chimarra sp au | 9 | 9.92 | 1.38 | 0.68 | 0.03 | 7 | 33.44 | 0.83 | 0.71 | 0.02 | 16 | 23.52 | 2.21 | 0.695 | 0.05 |
| QLD | Tropical | 364 | E | Baetidae | Offadens | Offadens o hickmani complex qld | 9 | 7.27 | 1.01 | 0.66 | 0.06 | 8 | 34.36 | 1.16 | 0.64 | 0.04 | 17 | 27.09 | 2.17 | 0.65 | 0.1 |
| QLD | Tropical | 364 | E | Leptophlebiidae | Atalomicria | Atalomicria sexfasciata sp A | 6 | 7.63 | 0.69 | 1.17 | 0.19 | 9 | 34.87 | 0.86 | 0.97 | 0.16 | 15 | 27.24 | 1.55 | 1.07 | 0.35 |
| QLD | Tropical | 364 | E | Leptophlebiidae | Austrophlebioides | Austrophlebioides rieki | 7 | 7.5 | 0.37 | 1.5 | 0.1 | 8 | 35.27 | 1.15 | 1.48 | 0.12 | 15 | 27.77 | 1.52 | 1.49 | 0.22 |
| QLD | Tropical | 364 | E | Leptophlebiidae | Austrophlebioides | Austrophlebioides rieki sp | 3 | 7.9 | 0.95 | 1.76 | 0.27 | 6 | 35.57 | 0.65 | 1.64 | 0.19 | 9 | 27.67 | 1.6 | 1.7 | 0.46 |
| QLD | Tropical | 364 | E | Leptophlebiidae | Nousia | Nousia sp A | NA | NA | NA | NA | NA | 5 | 34.56 | 0.8 | 1.12 | 0.14 | NA | NA | NA | NA | NA |
| QLD | Tropical | 364 | E | Leptophlebiidae | Nousia | Nousia sp B | 9 | 6.33 | 1.13 | 1.77 | 1.43 | 12 | 35.06 | 0.91 | 1.23 | 0.12 | 21 | 28.73 | 2.04 | 1.5 | 1.55 |
| QLD | Tropical | 364 | E | Leptophlebiidae | Nousia | Nousia sp C | 7 | 5.8 | 0.45 | 1.32 | 0.09 | 3 | 34.37 | 0.4 | 1.31 | 0.03 | 10 | 28.57 | 0.85 | 1.315 | 0.12 |
| QLD | Tropical | 364 | P | Eustheniidae | Cosmioperla | Cosmioperla kuna qld | 10 | 4.25 | 2.06 | 2.71 | 0.85 | 10 | 36.09 | 0.47 | 2.66 | 0.74 | 20 | 31.84 | 2.53 | 2.685 | 1.59 |
| QLD | Tropical | 364 | P | Gripopterygidae | Illisioperla | Illisioperla barbara | 10 | 10.17 | 2.71 | 1.21 | 0.44 | 5 | 33.26 | 1.68 | 1.05 | 0.2 | 15 | 23.09 | 4.39 | 1.13 | 0.64 |
| QLD | Tropical | 364 | P | Gripopterygidae | Illisioperla | Illisioperla cerberus | NA | NA | NA | NA | NA | 6 | 33.27 | 1.12 | 0.92 | 0.28 | NA | NA | NA | NA | NA |
| QLD | Tropical | 364 | T | Calamoceratidae | Anisocentropus | Anisocentropus sp qld | NA | NA | NA | NA | NA | 10 | 34.64 | 1.31 | 0.72 | 0.15 | NA | NA | NA | NA | NA |
| QLD | Tropical | 364 | T | Hydropsychidae | Asmicridea | Asmicridea sp C | 4 | 7.45 | 1.51 | 0.86 | 0.14 | 5 | 35.9 | 0.47 | 0.85 | 0.12 | 9 | 28.45 | 1.98 | 0.855 | 0.26 |
| QLD | Tropical | 364 | T | Hydropsychidae | Asmicridea | Asmicridea sp D | 9 | 7.03 | 1.2 | 0.94 | 0.13 | 10 | 35.86 | 0.99 | 0.91 | 0.16 | 19 | 28.83 | 2.19 | 0.925 | 0.29 |
| QLD | Tropical | 364 | T | Hydropsychidae | Asmicridea | Asmicridea sp E | 5 | 7.98 | 1.38 | 0.8 | 0.02 | 3 | 36.4 | 0.2 | 0.79 | 0.02 | 8 | 28.42 | 1.58 | 0.795 | 0.04 |
| QLD | Tropical | 364 | T | Odontoceridae | Marilia | Marilia bola | NA | NA | NA | NA | NA | 3 | 36.07 | 0.76 | 0.53 | 0.11 | NA | NA | NA | NA | NA |
| QLD | Tropical | 364 | T | Philopotamidae | Chimarra | Chimarra sp au | 3 | 8.7 | 0.5 | 0.65 | 0.05 | NA | NA | NA | NA | NA | NA | NA | NA | NA | NA |
| QLD | Tropical | 364 | T | Philopotamidae | Chimarra | Chimarra sp bt | NA | NA | NA | NA | NA | 3 | 34.03 | 0.4 | 0.75 | 0.02 | NA | NA | NA | NA | NA |
| QLD | Tropical | 364 | T | Philopotamidae | Chimarra | Chimarra sp du2t | 3 | 8.67 | 0.15 | 0.83 | 0.06 | NA | NA | NA | NA | NA | NA | NA | NA | NA | NA |
| QLD | Tropical | 364 | T | Philopotamidae | Chimarra | Chimarra sp tn | NA | NA | NA | NA | NA | 3 | 34.27 | 0.32 | 0.79 | 0.02 | NA | NA | NA | NA | NA |
| QLD | Tropical | 445 | E | Ameletopsidae | Mirawara | Mirawara qld | 3 | 5.53 | 0.06 | 3.64 | 0.5 | 3 | 32.67 | 1.95 | 2.28 | 0.55 | 6 | 27.14 | 2.01 | 2.96 | 1.05 |
| QLD | Tropical | 445 | E | Baetidae | Offadens | Offadens o hickmani complex qld | 3 | 9.7 | 1.21 | 1.73 | 1.63 | NA | NA | NA | NA | NA | NA | NA | NA | NA | NA |
| QLD | Tropical | 445 | E | Leptophlebiidae | Atalomicria | Atalomicria sexfasciata sp A | 11 | 7.25 | 0.7 | 1.23 | 0.32 | 10 | 32.2 | 2.77 | 1.01 | 0.13 | 21 | 24.95 | 3.47 | 1.12 | 0.45 |
| QLD | Tropical | 445 | E | Leptophlebiidae | Austrophlebioides | Austrophlebioides rieki | 4 | 8.22 | 0.49 | 1.7 | 0.29 | NA | NA | NA | NA | NA | NA | NA | NA | NA | NA |
| QLD | Tropical | 445 | E | Leptophlebiidae | Nousia | Nousia sp A | 4 | 6.97 | 0.84 | 1.49 | 0.07 | 7 | 33.63 | 1.39 | 1.04 | 0.11 | 11 | 26.66 | 2.23 | 1.265 | 0.18 |
| QLD | Tropical | 445 | E | Leptophlebiidae | Nousia | Nousia sp B | 7 | 7.89 | 1.26 | 1.3 | 0.21 | 6 | 33.37 | 1.71 | 1.09 | 0.24 | 13 | 25.48 | 2.97 | 1.195 | 0.45 |
| QLD | Tropical | 445 | E | Leptophlebiidae | Nousia | Nousia sp C | 3 | 7.77 | 1.15 | 1.42 | 0.19 | 4 | 34.17 | 0.33 | 1.2 | 0.1 | 7 | 26.4 | 1.48 | 1.31 | 0.29 |
| QLD | Tropical | 445 | P | Eustheniidae | Cosmioperla | Cosmioperla kuna qld | 12 | 4.29 | 2.05 | 2.49 | 0.82 | 11 | 34.45 | 1.23 | 2.16 | 0.84 | 23 | 30.16 | 3.28 | 2.325 | 1.66 |
| QLD | Tropical | 445 | P | Gripopterygidae | Illisioperla | Illisioperla barbara | 8 | 9.12 | 2.12 | 1.2 | 0.14 | 7 | 34.1 | 0.72 | 1.3 | 0.14 | 15 | 24.98 | 2.84 | 1.25 | 0.28 |
| QLD | Tropical | 445 | P | Gripopterygidae | Illisioperla | Illisioperla cerberus | 3 | 4.7 | 0.66 | 1.37 | 0.08 | 4 | 32.9 | 1.8 | 1.34 | 0.35 | 7 | 28.2 | 2.46 | 1.355 | 0.43 |
| QLD | Tropical | 445 | T | Calamoceratidae | Anisocentropus | Anisocentropus sp qld | NA | NA | NA | NA | NA | 9 | 32.94 | 2.58 | 0.82 | 0.15 | NA | NA | NA | NA | NA |
| QLD | Tropical | 445 | T | Calocidae | Calocoides | Calocoides aquilona | 10 | 6.61 | 1.35 | 0.7 | 0.07 | 7 | 33.7 | 3.15 | 0.8 | 0.29 | 17 | 27.09 | 4.5 | 0.75 | 0.36 |
| QLD | Tropical | 445 | T | Hydrobiosidae | Apsilochorema | Apsilochorema obliquum | NA | NA | NA | NA | NA | 3 | 31.2 | 0.26 | 0.66 | 0.04 | NA | NA | NA | NA | NA |
| QLD | Tropical | 445 | T | Hydrobiosidae | Taschorema complex | Taschorema evansi qld | 8 | 5.14 | 1.58 | 0.74 | 0.21 | 7 | 31.99 | 0.9 | 0.84 | 0.27 | 15 | 26.85 | 2.48 | 0.79 | 0.48 |
| QLD | Tropical | 445 | T | Hydropsychidae | Asmicridea | Asmicridea sp C | 5 | 5.58 | 0.72 | 1.08 | 0.12 | 3 | 34.13 | 1.59 | 1.12 | 0.09 | 8 | 28.55 | 2.31 | 1.1 | 0.21 |
| QLD | Tropical | 445 | T | Hydropsychidae | Asmicridea | Asmicridea sp D | 11 | 5.83 | 1.27 | 1.06 | 0.05 | 13 | 34.51 | 2.13 | 1.05 | 0.04 | 24 | 28.68 | 3.4 | 1.055 | 0.09 |
| QLD | Tropical | 445 | T | Hydropsychidae | Diplectrona | Diplectrona sp AV3 | 5 | 6.06 | 1.21 | 1.22 | 0.07 | 6 | 31.65 | 3.22 | 1.18 | 0.09 | 11 | 25.59 | 4.43 | 1.2 | 0.16 |
| QLD | Tropical | 445 | T | Leptoceridae | Triplectides | Triplectides australicus | 9 | 5.7 | 0.43 | 0.76 | 0.18 | 9 | 33.97 | 1.82 | 0.78 | 0.11 | 18 | 28.27 | 2.25 | 0.77 | 0.29 |
| QLD | Tropical | 445 | T | Leptoceridae | Triplectides | Triplectides gonetalus sp | 5 | 8.46 | 0.84 | 0.59 | 0.15 | 6 | 32.8 | 1.32 | 0.69 | 0.12 | 11 | 24.34 | 2.16 | 0.64 | 0.27 |
| QLD | Tropical | 445 | T | Odontoceridae | Barynema | Barynema sp AV1 | 6 | 5.33 | 0.23 | 1.47 | 0.28 | 6 | 33.8 | 1.08 | 1.56 | 0.25 | 12 | 28.47 | 1.31 | 1.515 | 0.53 |
| QLD | Tropical | 445 | T | Odontoceridae | Marilia | Marilia bola | 11 | 9.46 | 2.49 | 0.63 | 0.03 | 10 | 32.36 | 4.86 | 0.64 | 0.03 | 21 | 22.9 | 7.35 | 0.635 | 0.06 |
| QLD | Tropical | 445 | T | Philopotamidae | Chimarra | Chimarra sp bt | 12 | 8.58 | 0.84 | 0.73 | 0.04 | 11 | 29.26 | 1.47 | 0.73 | 0.04 | 23 | 20.68 | 2.31 | 0.73 | 0.08 |
| QLD | Tropical | 445 | T | Philorheithridae | Genus B | Genus B sp | 4 | 7.5 | 1.72 | 0.49 | 0.07 | 5 | 33.38 | 0.98 | 0.57 | 0.14 | 9 | 25.88 | 2.7 | 0.53 | 0.21 |
| QLD | Tropical | 705 | E | Ameletopsidae | Mirawara | Mirawara qld | 9 | 5.68 | 0.56 | 3.92 | 0.4 | 9 | 34.69 | 0.86 | 3.76 | 0.52 | 18 | 29.01 | 1.42 | 3.84 | 0.92 |
| QLD | Tropical | 705 | E | Baetidae | Offadens | Offadens o hickmani complex qld | 7 | 8.34 | 1.39 | 0.77 | 0.05 | 6 | 33.1 | 1.16 | 0.77 | 0.1 | 13 | 24.76 | 2.55 | 0.77 | 0.15 |
| QLD | Tropical | 705 | E | Leptophlebiidae | Austrophlebioides | Austrophlebioides rieki | NA | NA | NA | NA | NA | 5 | 33.4 | 1.04 | 1.56 | 0.43 | NA | NA | NA | NA | NA |
| QLD | Tropical | 705 | E | Leptophlebiidae | Nousia | Nousia sp A | 3 | 8.23 | 1.3 | 0.95 | 0.27 | 3 | 31.7 | 2.62 | 1.23 | 0.19 | 6 | 23.47 | 3.92 | 1.09 | 0.46 |
| QLD | Tropical | 705 | E | Leptophlebiidae | Nousia | Nousia sp B | 14 | 6.73 | 0.7 | 1.26 | 0.48 | 8 | 33.59 | 0.69 | 1.23 | 0.32 | 22 | 26.86 | 1.39 | 1.245 | 0.8 |
| QLD | Tropical | 705 | E | Leptophlebiidae | Nousia | Nousia sp C | 3 | 5.27 | 0.58 | 1.33 | 0.05 | 4 | 32.45 | 1.83 | 1.39 | 0.03 | 7 | 27.18 | 2.41 | 1.36 | 0.08 |
| QLD | Tropical | 705 | P | Gripopterygidae | Illisioperla | Illisioperla barbara | 7 | 7.26 | 2.81 | 0.87 | 0.34 | 9 | 33.57 | 0.53 | 1.02 | 0.32 | 16 | 26.31 | 3.34 | 0.945 | 0.66 |
| QLD | Tropical | 705 | P | Gripopterygidae | Illisioperla | Illisioperla sp qld | 13 | 3.54 | 1.61 | 0.71 | 0.07 | 11 | 32.55 | 1.88 | 0.69 | 0.12 | 24 | 29.01 | 3.49 | 0.7 | 0.19 |
| QLD | Tropical | 705 | T | Calamoceratidae | Anisocentropus | Anisocentropus sp qld | NA | NA | NA | NA | NA | 8 | 31.3 | 1.77 | 0.69 | 0.21 | NA | NA | NA | NA | NA |
| QLD | Tropical | 705 | T | Calocidae | Calocoides | Calocoides aquilona | 7 | 7.93 | 1.54 | 0.72 | 0.05 | 6 | 34.57 | 0.41 | 0.71 | 0.04 | 13 | 26.64 | 1.95 | 0.715 | 0.09 |
| QLD | Tropical | 705 | T | Glossosomatidae | Agapetus | Agapetus sp AV1 qld | 9 | 9.94 | 0.23 | 0.39 | 0.02 | 9 | 31.5 | 2.2 | 0.38 | 0.02 | 18 | 21.56 | 2.43 | 0.385 | 0.04 |
| QLD | Tropical | 705 | T | Helicophidae | Genus Cal Hel A | Genus Cal Hel A sp AV2 | 4 | 6.4 | 0.78 | 0.97 | 0.3 | NA | NA | NA | NA | NA | NA | NA | NA | NA | NA |
| QLD | Tropical | 705 | T | Hydrobiosidae | Taschorema complex | Taschorema evansi qld | 8 | 6.35 | 2.23 | 0.87 | 0.3 | 9 | 31.01 | 1.44 | 0.9 | 0.18 | 17 | 24.66 | 3.67 | 0.885 | 0.48 |
| QLD | Tropical | 705 | T | Hydropsychidae | Asmicridea | Asmicridea sp D | 5 | 5.1 | 0.69 | 1.09 | 0.07 | 7 | 34.5 | 1.21 | 1.09 | 0.06 | 12 | 29.4 | 1.9 | 1.09 | 0.13 |
| QLD | Tropical | 705 | T | Hydropsychidae | Diplectrona | Diplectrona sp AV3 | 6 | 6.02 | 0.82 | 1.29 | 0.29 | 4 | 33.92 | 2.81 | 1.29 | 0.3 | 10 | 27.9 | 3.63 | 1.29 | 0.59 |
| QLD | Tropical | 705 | T | Leptoceridae | Triplectides | Triplectides australicus | 3 | 5.73 | 2.03 | 0.37 | 0.11 | 6 | 34.53 | 0.43 | 0.39 | 0.09 | 9 | 28.8 | 2.46 | 0.38 | 0.2 |
| QLD | Tropical | 705 | T | Odontoceridae | Barynema | Barynema sp AV1 | NA | NA | NA | NA | NA | 4 | 33.45 | 1.62 | 1.25 | 0.28 | NA | NA | NA | NA | NA |
| QLD | Tropical | 705 | T | Philopotamidae | Chimarra | Chimarra sp au | 6 | 8.82 | 0.32 | 0.67 | 0.05 | 9 | 32.7 | 0.84 | 0.66 | 0.02 | 15 | 23.88 | 1.16 | 0.665 | 0.07 |
| QLD | Tropical | 705 | T | Philorheithridae | Aphilorheithrus | Aphilorheithrus sp AV3 | NA | NA | NA | NA | NA | 3 | 33.8 | 0.62 | 1.51 | 0.19 | NA | NA | NA | NA | NA |
| QLD | Tropical | 949 | E | Ameletopsidae | Mirawara | Mirawara qld | 3 | 5.13 | 0.4 | 3.62 | 0.71 | 3 | 34.23 | 0.49 | 2.78 | 0.32 | 6 | 29.1 | 0.89 | 3.2 | 1.03 |
| QLD | Tropical | 949 | E | Leptophlebiidae | Atalomicria | Atalomicria sexfasciata sp A | 4 | 8.62 | 1.48 | 0.93 | 0.08 | 3 | 32.1 | 0.53 | 1.02 | 0.14 | 7 | 23.48 | 2.01 | 0.975 | 0.22 |
| QLD | Tropical | 949 | E | Leptophlebiidae | Atalomicria | Atalomicria sexfasciata sp B | NA | NA | NA | NA | NA | 5 | 33.54 | 0.7 | 1.54 | 0.23 | NA | NA | NA | NA | NA |
| QLD | Tropical | 949 | E | Leptophlebiidae | Atalophlebia | Atalophlebia sp AV13 | 5 | 5.38 | 0.64 | 2.16 | 0.2 | 6 | 34.83 | 1.5 | 2.31 | 0.26 | 11 | 29.45 | 2.14 | 2.235 | 0.46 |
| QLD | Tropical | 949 | E | Leptophlebiidae | Nousia | Nousia sp | 3 | 7.37 | 2.29 | 1.3 | 0.01 | 6 | 33.53 | 0.29 | 1.21 | 0.11 | 9 | 26.16 | 2.58 | 1.255 | 0.12 |
| QLD | Tropical | 949 | E | Leptophlebiidae | Nousia | Nousia sp A | 5 | 8.42 | 2.11 | 0.98 | 0.14 | 6 | 32.37 | 1.12 | 1.13 | 0.17 | 11 | 23.95 | 3.23 | 1.055 | 0.31 |
| QLD | Tropical | 949 | E | Leptophlebiidae | Nousia | Nousia sp B | 16 | 7.5 | 1.18 | 1.09 | 0.19 | 5 | 33.22 | 0.88 | 1.18 | 0.15 | 21 | 25.72 | 2.06 | 1.135 | 0.34 |
| QLD | Tropical | 949 | P | Eustheniidae | Cosmioperla | Cosmioperla kuna qld | 4 | 4.18 | 0.33 | 2.17 | 0.7 | 3 | 34.93 | 0.81 | 1.68 | 0.56 | 7 | 30.75 | 1.14 | 1.925 | 1.26 |
| QLD | Tropical | 949 | P | Gripopterygidae | Illisioperla | Illisioperla barbara | 7 | 8.9 | 2.15 | 1.23 | 0.32 | 3 | 33.13 | 1.05 | 1.12 | 0.33 | 10 | 24.23 | 3.2 | 1.175 | 0.65 |
| QLD | Tropical | 949 | P | Gripopterygidae | Illisioperla | Illisioperla sp qld | NA | NA | NA | NA | NA | 4 | 33.1 | 1.25 | 0.67 | 0.18 | NA | NA | NA | NA | NA |
| QLD | Tropical | 949 | T | Calamoceratidae | Anisocentropus | Anisocentropus sp qld | NA | NA | NA | NA | NA | 9 | 32.23 | 1.92 | 0.78 | 0.14 | NA | NA | NA | NA | NA |
| QLD | Tropical | 949 | T | Calocidae | Calocoides | Calocoides aquilona | 8 | 7.75 | 1.56 | 0.75 | 0.08 | 8 | 35.32 | 0.41 | 0.79 | 0.08 | 16 | 27.57 | 1.97 | 0.77 | 0.16 |
| QLD | Tropical | 949 | T | Helicophidae | Genus Cal Hel A | Genus Cal Hel A sp AV2 | 8 | 9.38 | 2.61 | 1.11 | 0.28 | 6 | 34.22 | 3.08 | 1.18 | 0.39 | 14 | 24.84 | 5.69 | 1.145 | 0.67 |
| QLD | Tropical | 949 | T | Hydrobiosidae | Taschorema complex | Taschorema evansi qld | 8 | 6.61 | 3.24 | 0.81 | 0.25 | 9 | 30.98 | 1.13 | 0.92 | 0.23 | 17 | 24.37 | 4.37 | 0.865 | 0.48 |
| QLD | Tropical | 949 | T | Hydropsychidae | Asmicridea | Asmicridea sp B | NA | NA | NA | NA | NA | 3 | 34.8 | 0.36 | 1.08 | 0.26 | NA | NA | NA | NA | NA |
| QLD | Tropical | 949 | T | Hydropsychidae | Diplectrona | Diplectrona sp AV3 | 12 | 7.47 | 0.72 | 1.4 | 0.14 | 12 | 31.07 | 2.94 | 1.34 | 0.2 | 24 | 23.6 | 3.66 | 1.37 | 0.34 |
| QLD | Tropical | 949 | T | Leptoceridae | Triplectides | Triplectides australicus | 5 | 7.06 | 2.82 | 1 | 0.31 | NA | NA | NA | NA | NA | NA | NA | NA | NA | NA |
| QLD | Tropical | 949 | T | Leptoceridae | Triplectides | Triplectides gonetalus sp | 4 | 10.25 | 2.12 | 0.74 | 0.14 | 6 | 30.93 | 2.43 | 0.7 | 0.17 | 10 | 20.68 | 4.55 | 0.72 | 0.31 |
| QLD | Tropical | 949 | T | Odontoceridae | Barynema | Barynema sp AV1 | 12 | 8.77 | 2.46 | 1.25 | 0.5 | 12 | 31.68 | 2.27 | 1.17 | 0.4 | 24 | 22.91 | 4.73 | 1.21 | 0.9 |
| QLD | Tropical | 949 | T | Philopotamidae | Chimarra | Chimarra sp au | 7 | 9.04 | 1.34 | 0.64 | 0.02 | 4 | 32.77 | 0.85 | 0.65 | 0.05 | 11 | 23.73 | 2.19 | 0.645 | 0.07 |
| QLD | Tropical | 949 | T | Philopotamidae | Hydrobiosella | Hydrobiosella sp AV15 | NA | NA | NA | NA | NA | 6 | 33.25 | 0.46 | 0.76 | 0.03 | NA | NA | NA | NA | NA |

Supplementary Table S4. Mean and standard deviation (sd) of critical thermal maximum (CT_max_) and thermal breadth (*T*_br_) (both in °C) per (morpho)species and regions. The standard deviation (sd) of each species per region was used to assess intra-specific variation. The mean of each metric per species is used to calculate the inter-specific variation. Note that n refers to the number of individuals and N refers to the number of populations (sites in each region) where the indices were recorded on the species. E= Ephemeroptera, P=Plecoptera, T-Trichoptera. Our study sites in NSW are in a temperate location and those in QLD a tropical location.

| Region | Order | Species | Mean CTmax | Sd of CTmax | n (individuums) | Mean *T*br | Sd of *T*b | *N* (populations) |
| --- | --- | --- | --- | --- | --- | --- | --- | --- |
| NSW | E | Atalophlebia albiterminata | 34.99 | 2.76 | 14 | 32.57 | NA | 1 |
|  |  | Atalophlebia sp. AV2 | 34.73 | 5.29 | 4 | NA | NA | NA |
|  |  | Atalophlebia sp. AV21 | 32.40 | 1.61 | 3 | NA | NA | NA |
|  |  | Atalophlebia sp. AV4 | 32.46 | 2.01 | 11 | 32.50 | NA | 1 |
|  |  | Austrophlebioides marchanti | 32.87 | 1.68 | 18 | 28.87 | 0.78 | 2 |
|  |  | Austrophlebioides marchanti sp. | 31.61 | 1.32 | 25 | 28.88 | 0.87 | 4 |
|  |  | Austrophlebioides pusillus | 32.70 | 3.78 | 17 | 30.94 | 2.91 | 2 |
|  |  | Austrophlebioides sp. AV2 | 32.91 | 1.95 | 24 | 30.79 | 1.50 | 2 |
|  |  | Coloburiscoides giganteus | 32.51 | 2.39 | 37 | 30.56 | 2.23 | 4 |
|  |  | Coloburiscoides sp. | 33.02 | 1.20 | 30 | 30.28 | 0.31 | 3 |
|  |  | Garinjuga sp. AV1 | 34.23 | 1.65 | 6 | NA | NA | NA |
|  |  | Mirawara sp | 32.83 | 1.34 | 4 | NA | NA | NA |
|  |  | Nousia sp. AV1 | 33.06 | 1.54 | 15 | NA | NA | NA |
|  |  | Nousia sp. AV11 | 31.38 | 1.00 | 16 | 29.99 | NA | 1 |
|  |  | Nousia sp. AV2 | 30.30 | 2.00 | 4 | 29.53 | NA | 1 |
|  |  | Nousia spp AV11 | 31.42 | 0.94 | 26 | 29.22 | 1.01 | 2 |
|  |  | Offadens o hickmani complex | 29.56 | 2.31 | 107 | 26.12 | 1.83 | 9 |
|  | P | Austrocercella spp. | 30.75 | 0.21 | 4 | NA | NA | NA |
|  |  | Austroheptura sp. 2 | 32.85 | 1.16 | 21 | 31.76 | 0.65 | 3 |
|  |  | Cosmioperla kuna | 33.92 | 1.74 | 51 | 33.42 | 1.51 | 6 |
|  |  | Dinotoperla brevipennis | 31.64 | 1.93 | 5 | 26.47 | NA | 1 |
|  |  | Dinotoperla christinae | 30.86 | 2.40 | 21 | 29.62 | 2.06 | 2 |
|  |  | Dinotoperla eucumbene | 33.20 | 1.74 | 32 | 30.22 | 2.11 | 4 |
|  |  | Dinotoperla fontana | 33.76 | 1.42 | 12 | 30.80 | NA | 1 |
|  |  | Dinotoperla serricauda thwaitesi | 33.83 | 0.66 | 4 | NA | NA | NA |
|  |  | Eunotoperla sp. | 31.38 | 3.26 | 5 | NA | NA | NA |
|  |  | Riekoperla alpina | 33.27 | 0.49 | 3 | 32.35 | NA | 1 |
|  |  | Riekoperla rugosa group | 33.76 | 1.48 | 17 | 31.44 | 0.87 | 2 |
|  |  | Trinotoperla sp. | 31.14 | 2.89 | 28 | 27.12 | 4.61 | 3 |
|  | T | Anisocentropus sp. | 30.40 | 3.33 | 24 | NA | NA | NA |
|  |  | Aphilorheithrus sp. | 28.73 | 1.07 | 3 | NA | NA | NA |
|  |  | Aphilorheithrus spp. | 28.03 | 1.36 | 3 | NA | NA | NA |
|  |  | Aphilorheithrus spp. AV3 | 30.47 | 2.46 | 3 | NA | NA | NA |
|  |  | Apsilochorema obliquum | 27.73 | 1.78 | 3 | NA | NA | NA |
|  |  | Asmicridea sp. AV1 | 32.06 | 2.31 | 51 | 29.17 | 1.96 | 5 |
|  |  | Austrheithrus sp. | 33.43 | 1.53 | 3 | NA | NA | NA |
|  |  | Cheumatopsyche sp. AV3 | 33.32 | 1.36 | 12 | 31.53 | NA | 1 |
|  |  | Cheumatopsyche sp. AV4 | 36.57 | 0.55 | 3 | NA | NA | NA |
|  |  | Chimarra monticola | 31.45 | 2.05 | 44 | 27.71 | 1.67 | 5 |
|  |  | Coenoria sp. AV4 | 33.33 | 1.89 | 9 | 28.64 | NA | 1 |
|  |  | Conoesucus sp. AV2 | 32.70 | 2.61 | 37 | 28.70 | 2.10 | 4 |
|  |  | Diplectrona sp. | 28.43 | 3.35 | 3 | NA | NA | NA |
|  |  | Kosrheithrus tillyardi | 30.87 | 2.56 | 39 | 28.28 | 2.23 | 5 |
|  |  | Notalina fulva | 32.73 | 2.76 | 3 | 29.38 | NA | 1 |
|  |  | Tamasia acuta | 29.86 | 2.15 | 30 | 25.58 | 2.12 | 4 |
|  |  | Tamasia sp. AV1 | 31.24 | 1.95 | 14 | 26.76 | 1.05 | 2 |
|  |  | Taschorema evansi | 29.22 | 1.27 | 39 | 27.06 | 1.11 | 6 |
|  |  | Triplectides ciuskus ciuskus | 35.46 | 3.89 | 13 | 33.43 | NA | 1 |
|  |  | Triplectides proximus | 30.87 | 2.09 | 9 | NA | NA | NA |
|  |  | Triplectides proximus sp. | 30.69 | 1.13 | 7 | 25.24 | NA | 1 |
|  |  | Ulmerochorema rubiconum | 29.12 | 1.85 | 9 | 24.65 | NA | 1 |
| QLD | E | Atalomicria sexfasciata sp. A | 33.38 | 1.97 | 24 | 25.22 | 1.89 | 3 |
|  |  | Atalomicria sexfasciata sp. B | 33.54 | 0.70 | 5 | NA | NA | NA |
|  |  | Atalophlebia sp. AV13 | 34.83 | 1.50 | 6 | 29.45 | NA | 1 |
|  |  | Austrophlebioides rieki | 34.71 | 1.30 | 22 | 27.98 | 0.30 | 2 |
|  |  | Austrophlebioides rieki sp. | 35.31 | 0.98 | 16 | 27.97 | 0.42 | 2 |
|  |  | Austrophlebioides rieki spp. | 34.90 | 1.13 | 4 | 28.14 | NA | 1 |
|  |  | Mirawara qld | 34.19 | 1.29 | 15 | 28.42 | 1.11 | 3 |
|  |  | Nousia sp. | 33.53 | 0.29 | 6 | 26.16 | NA | 1 |
|  |  | Nousia sp. A | 33.44 | 1.58 | 24 | 24.69 | 1.72 | 3 |
|  |  | Nousia sp. B | 34.16 | 1.26 | 38 | 27.07 | 1.53 | 5 |
|  |  | Nousia sp. C | 33.94 | 1.28 | 15 | 27.76 | 1.17 | 4 |
|  |  | Offadens o hickmani complex qld | 33.55 | 1.16 | 21 | 25.83 | 1.18 | 3 |
|  | P | Cosmioperla kuna qld | 35.20 | 1.12 | 24 | 30.92 | 0.85 | 3 |
|  |  | Illisioperla barbara | 33.97 | 1.01 | 34 | 25.03 | 1.45 | 5 |
|  |  | Illisioperla cerberus | 33.64 | 1.50 | 14 | 28.20 | NA | 1 |
|  |  | Illisioperla sp. qld | 32.69 | 1.71 | 15 | 29.01 | NA | 1 |
|  |  | Trinotoperla sp. A | 33.58 | 2.22 | 12 | NA | NA | NA |
|  | T | Agapetus sp. AV1 qld | 31.50 | 2.20 | 9 | 21.56 | NA | 1 |
|  |  | Anisocentropus sp. qld | 33.32 | 2.25 | 46 | NA | NA | NA |
|  |  | Aphilorheithrus sp. AV3 | 33.80 | 0.62 | 3 | NA | NA | NA |
|  |  | Apsilochorema obliquum | 31.20 | 0.26 | 3 | NA | NA | NA |
|  |  | Asmicridea sp. B | 34.80 | 0.36 | 3 | NA | NA | NA |
|  |  | Asmicridea sp. C | 34.95 | 1.43 | 12 | 28.17 | 0.57 | 3 |
|  |  | Asmicridea sp. D | 34.86 | 1.66 | 44 | 28.45 | 1.09 | 4 |
|  |  | Asmicridea sp. E | 35.73 | 0.93 | 6 | 27.09 | 1.87 | 2 |
|  |  | Barynema sp. AV1 | 32.85 | 2.05 | 26 | 25.69 | 3.93 | 2 |
|  |  | Calocoides aquilona | 34.70 | 1.86 | 21 | 27.10 | 0.47 | 3 |
|  |  | Chimarra sp. au | 32.94 | 1.10 | 22 | 23.71 | 0.18 | 3 |
|  |  | Chimarra sp. bt | 30.55 | 2.53 | 15 | 20.68 | NA | 1 |
|  |  | Chimarra sp. du2t | 33.48 | 0.69 | 4 | NA | NA | NA |
|  |  | Chimarra sp. tn | 34.20 | 0.29 | 4 | NA | NA | NA |
|  |  | Diplectrona sp. AV3 | 31.75 | 3.05 | 22 | 25.70 | 2.15 | 3 |
|  |  | Genus B sp. | 33.42 | 0.88 | 6 | 25.88 | NA | 1 |
|  |  | Genus Cal Hel A sp. AV2 | 33.69 | 2.89 | 8 | 24.84 | NA | 1 |
|  |  | Hydrobiosella sp. AV15 | 33.19 | 0.46 | 8 | NA | NA | NA |
|  |  | Marilia bola | 33.22 | 4.52 | 13 | 22.90 | NA | 1 |
|  |  | Taschorema evansi qld | 31.35 | 1.19 | 25 | 25.29 | 1.36 | 3 |
|  |  | Triplectides australicus | 34.12 | 1.41 | 16 | 28.54 | 0.37 | 2 |
|  |  | Triplectides gonetalus | 34.07 | 1.00 | 3 | NA | NA | NA |
|  |  | Triplectides gonetalus sp. | 32.03 | 2.10 | 13 | 22.51 | 2.59 | 2 |


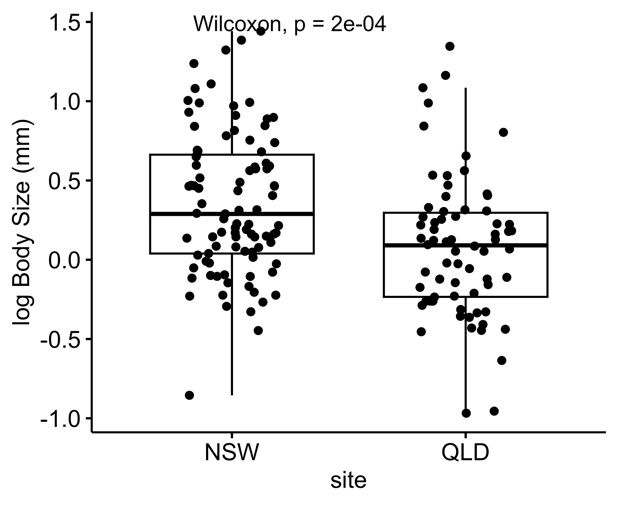


Supplementary Figure S2. Body size (log_10_ head width in mm) comparison for the two climate zones. Our study sites in NSW are in a temperate location and those in QLD a tropical location.

*Supplementary Table S5.* Summary linear mixed models for Thermal Breadth (*T*_br_), sorted from the strongest to the weakest model in terms of Akaike information criterion (AIC). Predictor variables were chosen based on ecological relevance and PCA (Supplementary Figure S5, see also Supplementary Tables S2 and S5). Random effects were ‘sampling site’ and the nested effect ‘family/genus/species’ (for the grey highlighted models), or ‘order/family/genus/species’. Df is the number of parameters in the model (default df = 2). LogLik (Log-Likelihood) describes how likely the model is. AICc is the information score of the model. ΔAIC is the difference between AICc score between the best model and the model being compared. Models with ΔAIC ≤ 2 have substantial support, which then lessens until with ΔAIC ≥ 10 no support is given to the model anymore. W_i_ (weight) is the proportion of the predictive power provided by the model. The conditional r^2^ takes both the fixed and random effects into account. The marginal r^2^ considers only the variance of the fixed effects. Taxonomic order refers to Ephemeroptera (E), Plecoptera (P) or Trichoptera (T).

Supplementary Table S6. Results of the second best fit mixed effect model, with 163 observations, 65 species, 31 genera, 17 families and 14 sampling sites.

| **Random effects** | Variance | Standard Deviation |
| --- | --- | --- |
| species:(genus:family) | 0.981 | 0.991 |
| genus:family | 0.542 | 0.736 |
| family | 0.095 | 0.308 |
| sampling site | 0.395 | 0.628 |
| residual | 2.338 | 1.529 |

| **Fixed Effects** | Estimate | Standard Error | *t*-Value |
| --- | --- | --- | --- |
| Intercept | 22.041 | 2.355 | 9.361 |
| Annual range | 0.158 | 0.043 | 3.646 |
| Annual max | 0.107 | 0.102 | 1.048 |
| Body size (log) | 2.094 | 0.546 | 3.839 |
| Contrast (E-P) | 1.827 | 0.754 | 2.421 |
| Contrast (E-T) | -0.424 | 0.687 | -0.617 |
| Contrast () | -2.251 | 0.696 | -3.233 |

| 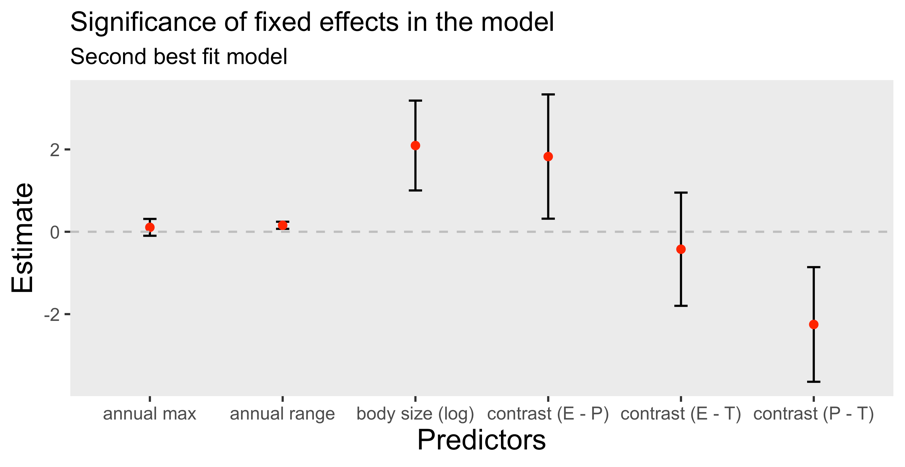  A | |
| --- | --- |
| B | B1 |
| 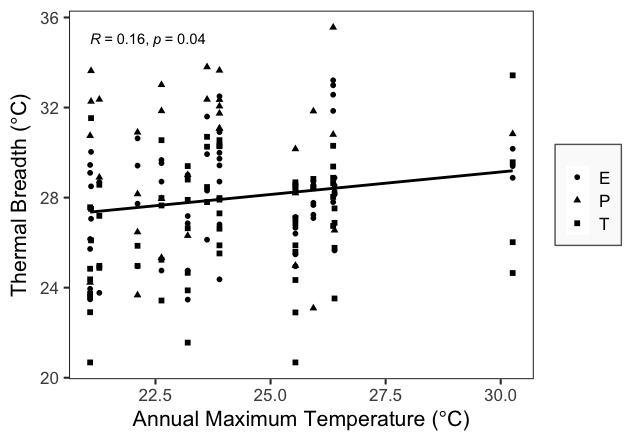 | 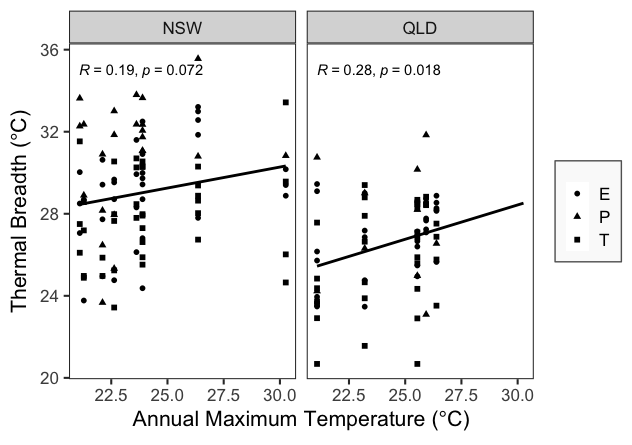 |
|  |  |

Supplementary Figure S3. (A) Results of the second-best fitted model. Annual maximum temperature is not significant predictor for Thermal Breadth, which supports the CVH. (B) Change of Thermal Breadth with increasing annual maximum temperature for all data, and (B1) for each climate zone in comparison. E= Ephemeroptera, P=Plecoptera, T-Trichoptera. Our study sites in NSW are in a temperate location and those in QLD a tropical location.

| **Supplementary Text 1: List of Taxonomic Keys and other resources used for identification in our study.** |
| --- |
| - Cairns, Andi; Davis, Linda; Pearson, Richard (2017): **Guide to the riffle invertebrates of Australian Wet Tropics streams with a bibliography of their ecology.** Centre for Tropical Water & Aquatic Ecosystem Research (TropWATER); Publication 17/09, James Cook University, Townsville; 41 pp.  - Cartwright, David I. (1998): **Preliminary guide to the identification of late instar larvae of Australian Ecnomidae, Philopotamidae and Tasmiidae (Insecta: Trichoptera).** Identification Guide No.10, Cooperative Research Centre for Freshwater Ecology *ISBN 1 876144 04 1;* *ISSN 1321-280X*  - Cartwright, David I. (1998): **Preliminary guide to the identification of late instar larvae of Australian Polycentropodidae, Glossomatidae, Dipsuedopsidae and Psychomyiidae (Insecta: Trichoptera).** Identification Guide No.15, Cooperative Research Centre for Freshwater Ecology *ISBN 1 876144 11 4;* *ISSN 1321-280X*  - Dean, John C.; Suter, Phillip J. (1996): **Mayfly nymphs of Australia: A guide to genera.** Identification Guide No.7, Cooperative Research Centre for Freshwater Ecology *ISBN 1 876144 01 7;* *ISSN 1321-280X*  - Dean, John C. (1999): **Larvae of the Australian Hydrobiosidae (Insecta: Trichoptera).** Identification Guide No.11, Cooperative Research Centre for Freshwater Ecology *ISBN 1 876144 08 4;* *ISSN 1321-280X*  - Dean, John C. (1999): **Preliminary keys for the identification of Australian Trichoptera larvae of the family Hydropsychidae.** Identification Guide No.22, Cooperative Research Centre for Freshwater Ecology *ISBN 1 876144 23 8;* *ISSN 1321-280X*  - Dean, John C.; St Clair, Rosalind M.; Cartwright, David I. (2004): **Identification keys to Australian families and genera of caddis-fly larvae (Trichoptera).** Identification & Ecology Guide No.50, Cooperative Research Centre for Freshwater Ecology *ISBN 1 876144 53 X;* *ISSN 1446-7852*  **- Identification and ecology of Australian freshwater invertebrates – An interactive guide with colour digital imagery to assist in the identification of aquatic invertebrates.** Centre for Freshwater Ecosystems. *https://www.mdfrc.org.au/bugguide/display.asp?type=2&class=17*  - Jackson, Jean (1998): **Preliminary guide to the identification of late instar larvae of Australian Calocidae, Helicophidae and Conoesucidae (Insecta: Trichoptera).** Identification Guide No.16, Cooperative Research Centre for Freshwater Ecology *ISBN 1 876144 12 2;* *ISSN 1321-280X*  - Mynott, Julia H. (2011): **DRAFT - Preliminary key to the larvae of *Riekoperla* (Plecoptera: Gripopterygidae).** La Trobe University, Wodonga & Taxonomic Research and Information Network (TRIN)  - Shackleton, Michael (2013): **Preliminary keys to the larvae of Australian Calocidae (Trichoptera: Insecta).** La Trobe University, Wodonga & Taxonomic Research and Information Network (TRIN)  - St Clair, Rosalind M. (1994): **Some larval Leptoceridae (Trichoptera) from south-eastern Australia.** Records of the Australian Museum. 46(2):171-226 *ISSN 0067-1975*  - St Clair, Rosalind M. (2000): **Preliminary guide to the identification of late instar larvae of Australian Philorheithridae, Calamoceratidae and Helicopsychidae (Insecta: Trichoptera).** Identification Guide No.12, Cooperative Research Centre for Freshwater Ecology *ISBN 1 876144 06 8; ISSN 1321-280X*  - St Clair, Rosalind M. (2000): **Preliminary keys for the identification of Australian caddisfly larvae of the family Leptoceridae.** Identification Guide No.27, Cooperative Research Centre for Freshwater Ecology *ISBN 1 876144 28 9; ISSN 1321-280X*  - St Clair, Rosalind M. (2000): **Preliminary keys for the identification of Australian caddisfly larvae of the family Odontoceridae, Kokiriidae and Oeconesidae.** Identification Guide No.30, Cooperative Research Centre for Freshwater Ecology *ISBN 1 876144 31 9; ISSN 1321-280X*  - Suter, Phillip J. (1997): **Preliminary guide to the identification of nymphs of Australian Baetid mayflies (Insecta: Ephemroptera) found in flowing waters.** Identification Guide No.14, Cooperative Research Centre for Freshwater Ecology *ISBN 1 876144 05 X;* *ISSN 1321-280X*  - Suter, John C. (1999): **Preliminary keys for the identification of Australian mayfly nymphs of the family Leptophlebiidae.** Identification Guide No.20, Cooperative Research Centre for Freshwater Ecology *ISBN 1 876144 21 1;* *ISSN 1321-280X*  - Suter, Phillip J. (1999): **Illustrated key to the Australian Caenid nymphs (Ephemeroptera: Caenidae).** Identification Guide No.23, Cooperative Research Centre for Freshwater Ecology *ISBN 1 876144 24 6;* *ISSN 1321-280X*  - Suter, Phillip J.; Webb, Jeff M.; Rowe, Darryl (2009): **Key to mature nymphs of *Coloburiscoides* (Lestage) (Ephemeroptera: Coloburiscidae).** Museum Victoria Science Reports. 14:1-24  - Webb, Jeff M.; Suter, Phillip J. (2011):**Identification of Larvae of Australian Baetidae.** Museum Victoria Science Reports. 15:1-24 *ISSN 1833-0290*  - Yule, Cathy (1997): **Identification guide to the stonefly nymphs of New South Wales and northern Victoria.** Australian Water Technology Identification Guide Number 2 *ISBN 07313 0290 3* |
